# Hypoxia or tobacco-smoke exposure induce region-specific microvascular remodeling in the brain

**DOI:** 10.1038/s41598-026-45975-3

**Published:** 2026-04-17

**Authors:** Nazli Salik Demirtas, Edma Loku, Yasmine Porschen, Julia Schäffer, Cheng-Yu Wu, Christoph Rummel, Natascha Sommer, Stefan Hadzic, Norbert Weissmann, Till Acker, Attila Németh

**Affiliations:** 1 Institute of Neuropathology, Justus Liebig University Giessen, Giessen, Germany; 2 Excellence Cluster Cardio-Pulmonary Institute (CPI), Universities of Giessen and Marburg Lung Center, Member of the German Center for Lung Research (DZL), Justus Liebig University Giessen, Giessen, Germany; 3 Center for Mind, Brain and Behavior (CMBB), University of Marburg and Justus Liebig University Giessen, Marburg, Germany; 4 Institute of Veterinary Physiology and Biochemistry, Justus Liebig University Giessen, Giessen, Germany; 5 Translational Neuroscience Network Giessen, Justus Liebig University Giessen, Giessen, Germany

**Keywords:** Brain microvascular remodeling, COPD, Hypoxia, Lung emphysema, Pulmonary hypertension, Tobacco-smoke, Diseases, Medical research, Neuroscience

## Abstract

Chronic obstructive pulmonary disease (COPD) leads to a progressive decline in lung function, making it a leading cause of mortality worldwide. While COPD primarily affects the lungs, emerging research shows the life-threatening role of associated systemic changes affecting other organs, including the brain. However, the complex interplay between COPD and the brain’s microenvironment remains poorly understood. Here, we monitored alterations across several brain regions in mouse models subjected to chronic mild hypoxia (CMH) or tobacco-smoke exposure leading to pulmonary hypertension (S-PH) or also emphysema (S-Em). Brain microvasculature, microglia and hypoxia pathway states were analyzed by immunofluorescence and immunohistochemistry imaging of standard FFPE tissue sections. Quantitative evaluation revealed model-specific changes in microvascular density, with increased density in the CMH and S-Em and decreased density in the S-PH mice. 3D-imaging of cleared thick brain tissue sections by spinning-disc confocal microscopy corroborated these findings and, together with fibrinogen, MMP-9 and TIMP-1 immunoblot analyses, provided a comprehensive view of the distinct microenvironment states. Overall, our findings link lung dysfunction and associated changes in the brain microenvironment, which may be relevant to understanding the systemic comorbidities of chronic lung diseases, particularly altered brain conditions that may influence lung cancer metastasis.

**Supplementary Information:**

The online version contains supplementary material available at 10.1038/s41598-026-45975-3.

## Introduction

Chronic obstructive pulmonary disease (COPD) is a debilitating lung disease characterized by long-term respiratory problems and obstruction of lung airflow, accompanied by systemic pathophysiological changes. It is estimated to be the third most common cause of death worldwide^1^. COPD is a mixed term comprising chronic bronchitis and airway obstruction as well as lung emphysema. Chronic lung diseases, such as COPD, can result in airway damage due to chronic inflammation and oxidative stress, particularly from inhaled irritants like cigarette smoke. This airway damage, however, leads to global alveolar hypoxia only in end-stage COPD patients^2^. Smoking is a known risk factor for the development of COPD and it is associated with increased mortality in lung cancer brain metastases^3–6^. Both tobacco-smoke-induced oxidative and inflammatory stress and brain-tissue-hypoxia may contribute to creating a supportive microenvironment for brain-related comorbidities of chronic lung diseases, such as lung cancer brain metastases. While previous studies have identified mechanistic links between COPD and lung cancer^3,7,8^, the development of COPD- and other chronic lung disease-associated tissue remodeling in the brain microenvironment remains largely unclear. Understanding these co-evolving changes and their interdependence is critical, as they influence patient survival and could lead to new therapeutic targets.

Systemic hypoxia is a reduced supply of oxygen to the tissues that triggers adaptive cellular mechanisms in the affected tissues via the oxygen sensing pathway. Over the last few decades, detailed studies of molecular mechanisms of cellular oxygen sensing have elucidated the central role of a hypoxia signaling pathway, in which hypoxia-inducible factors (HIFs) act as transcription factors and key regulators of gene expression programs^9^. Recent research suggests additional molecular oxygen sensing mechanisms through epigenetic regulatory enzymes, specific (histone) lysine demethylases (KDMs), which may act in concert with HIFs in adaptive gene regulation^10,11^. An important consequence of the hypoxia response at the tissue level is microvascular remodeling, which appears to be particularly pronounced in the brain^12^. Recent studies, largely based on experiments with a chronic mild hypoxia mouse model, indicate that systemic hypoxia causes extensive microvascular remodeling in the brain and suggest a supportive role for microglia in this process^12–15^.

Regarding potential mediators of brain microvascular remodeling, fibrinogen can induce endothelial cell migration and permeability as well as changes in tight junction proteins, as shown in cell culture studies^16–18^. Such changes also apply to the brain, where elevated levels of fibrinogen in the blood contribute to cerebrovascular remodeling via local activation of matrix metalloproteinase 9 (MMP-9)^19–21^. In addition, a recent study suggests that the age-related increase in fibrinogen levels leads to blood-brain barrier dysregulation via a dynamin-related protein 1-dependent pathway^22^. Fibrinogen can play various roles in the central nervous system, as summarized in a review by Petersen and colleagues^23^. At the cellular level, fibrinogen has been shown to regulate the function of endothelial and microglial cells as well as oligodendrocyte precursor cells and astrocytes^23–27^. Importantly, fibrinogen is a well-established systemic inflammation marker of COPD^28,29^. Fibrinogen levels are also elevated during long-term hypoxia in humans, whereas short-term hypoxia in mice does not cause systemic changes in fibrinogen levels^30^.

In this study, we systematically characterized microvascular and microglial changes in mouse models of chronic mild hypoxia (CMH) compared to tobacco-smoke-induced pulmonary hypertension (S-PH) and tobacco-smoke-induced PH plus emphysema (S-Em)^31^, investigated hypoxia and vascular leakage markers in the brain microenvironment of these models and explored the potential contribution of fibrinogen and MMP-9 to microvascular remodeling in the brain. We show that the most prominent microvascular changes occur upon tobacco-smoke-induced stress in the hippocampus and cortical regions. To our knowledge, this study represents the first systematic exploratory mapping of the brain microenvironment remodeling in lung disease models, comparing hypoxic (CMH) and inflammatory/oxidative stressors (S-PH/S-Em).

## Results

### Specific hypoxia and tobacco-smoke-induced changes in respiratory and cardiovascular function in the CMH, S-PH and S-Em mouse models

**Fig. 1:**
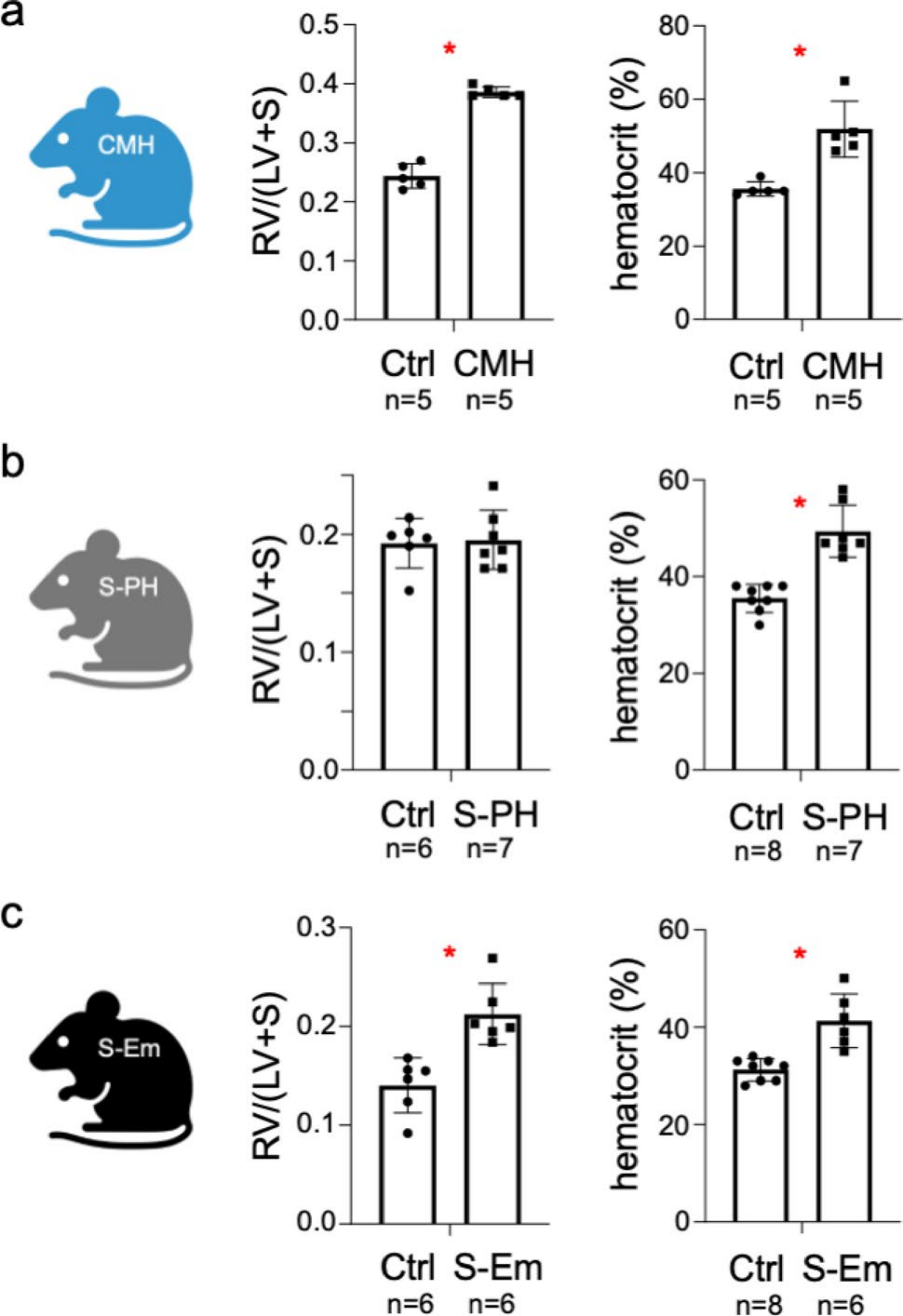
Cardiovascular changes in mouse models of chronic mild hypoxia and smoke exposure. Measurements (**a**) in the CMH model (10% O_2_ for 28 days), (**b**) in the S-PH model (cigarette smoke exposure for 3 months) and (**c**) in the S-Em model (cigarette smoke exposure for 8 months). Left: weight ratio of the right ventricle (RV) to the left ventricle with septum (LV + S). Note that no significant changes in LV + S mass were observed between the groups analyzed. Right: hematocrit levels. Unpaired t-tests *p* < 0.05 are marked with a red asterisk. The illustrations in all Figures were created with BioRender.com.

To monitor characteristic physiological changes in the mouse models for CMH, S-PH and S-Em, we first measured the weight ratio of the right ventricle to the left ventricle with septum and hematocrit levels. Right ventricular hypertrophy was only observed in the CMH and S-Em models, while this adaptation was not detected in the S-PH model (Fig. 1a–c). The significantly increased hematocrit values reflected hypoxemia in the CMH mice whereas the increase in hematocrit upon smoke exposure is most likely caused by the smoke itself (e.g. CO)^32,33^, as it was previously shown that our smoke-exposed mice are not hypoxic^31^(Fig. 1a–c). To characterize the smoke-exposure mouse model in more detail, hemodynamic measurements and concomitant heart rate recordings were performed. Right ventricular systolic pressure significantly increased in both the S-PH and S-Em mice compared to controls, while left ventricular systolic pressure, mean arterial pressure and heart rate remained unchanged (Supplementary Figure S1a, b). Finally, the development of emphysema in the S-Em mice was confirmed by evaluating dynamic and static lung compliance (Supplementary Figure S1b). Overall, the results comprehensively demonstrated the successful induction of characteristic systemic changes in the mouse models.

### Microvascular remodeling is brain region-specific and varies between the CMH, tobacco-smoke-induced S-PH and S-Em models

**Fig. 2:**
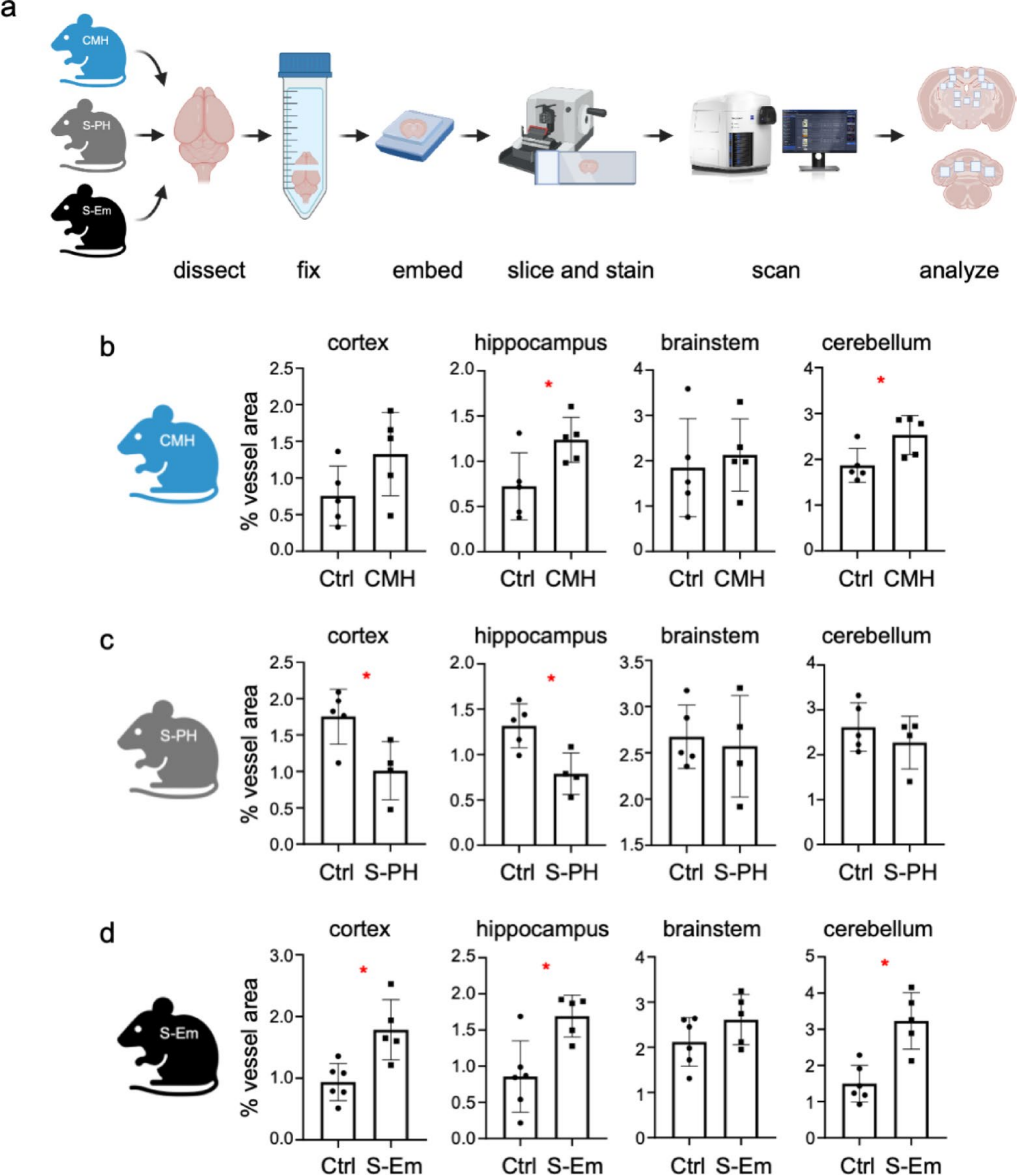
Microvascular densities of different brain areas in mouse models of chronic mild hypoxia and smoke exposure. (**a**) Workflow scheme of IF analyses of 5 μm FFPE tissue sections. The selected ROIs from coronal sections of the cortex, hippocampus, brainstem and cerebellum (from top to bottom) are shown on the right. Quantitative evaluation of endothelial marker podocalyxin IF staining (**b**) in the CMH model (*n* = 5 mice for both control and for the cohort treated with 10% O_2_ for 28 days), (**c**) in the S-PH model (*n* = 5 mice for the control and *n* = 4 mice for the cohort treated with cigarette smoke for 3 months) and (**d**) in the S-Em model (*n* = 6 mice for the control and *n* = 5 mice for the cohort treated with cigarette smoke for 8 months). Unpaired t-tests *p* < 0.05 are marked with a red asterisk.

Next, we analyzed the adaptive changes in the microvasculature of the brain caused by hypoxia and tobacco-smoke-induced lung injury. Brain FFPE tissue sections were subjected to immunofluorescence staining of the endothelial marker podocalyxin (see Supplementary Figure S2 for representative images). Several areas from the cortex, hippocampus, brainstem and cerebellum were selected for analysis (Fig. 2a) and the microvascular densities were calculated as a percentage of vessel area. Evaluation of CMH tissue samples revealed that after the initial phase of microvascular remodeling (4–14 days^15)^, after 28 days the brain exhibited region-specific remodeled microvascular structures. Thus, a significantly increased microvascular density in the hippocampus and cerebellum was observed after 28 days of hypoxia treatment (Fig. 2b). Interestingly, the S-PH model after 3 months of tobacco-smoke-induced lung damage demonstrated a marked decrease in microvascular density in the cortex and hippocampus, with other brain regions showing a similar trend for decreased microvascular density that did not reach statistical significance (Fig. 2c). Conversely, S-Em brains adapted to 8 months of tobacco-smoke-induced lung injury showed increased microvascular density in the cortex, hippocampus and cerebellum (Fig. 2d), similar to the CMH model. The results of the individual ROI analyses (Supplementary Table S1) indicate local differences in microvascular density, which become hidden in the cumulative evaluations of larger anatomical areas shown in (Fig. 2b–d). Taken together, these findings clearly show that the adaptive changes in the brain result in region-specific increased microvascular density increases in the CMH, S-Em and decreases in the S-PH model.

### Microglia density and distribution as well as HIF levels remain largely unaffected in the CMH, S-PH and S-Em models

**Fig. 3:**
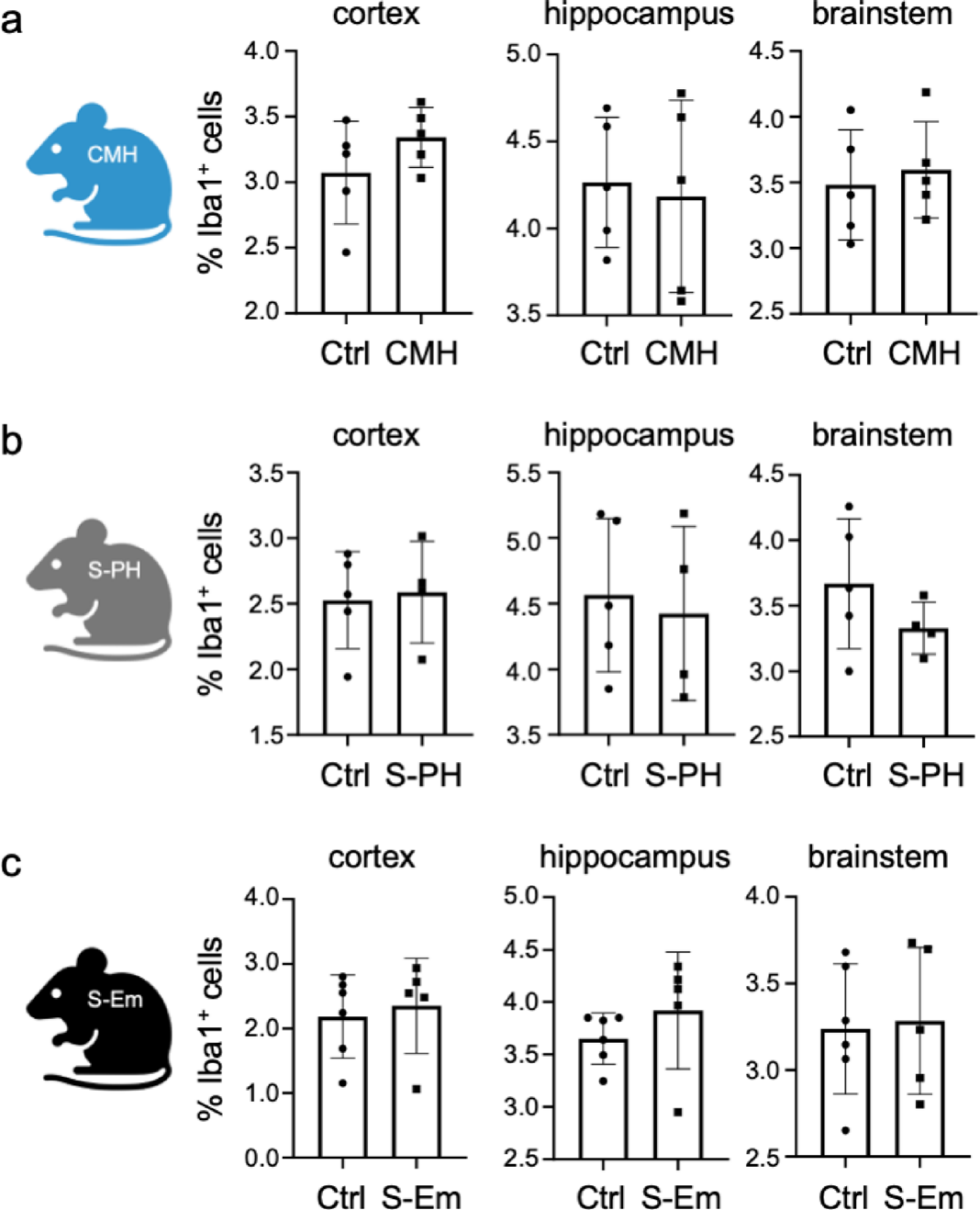
Microglia densities of different brain areas in mouse models of chronic mild hypoxia and smoke exposure. Quantitative evaluation of microglia/macrophage marker Iba1 immunofluorescence staining (**a**) in the CMH model (*n* = 5 mice for both control and for the cohort treated with 10% O_2_ for 28 days), (**b**) in the S-PH model (*n* = 5 mice for the control and *n* = 4 mice for the cohort treated with cigarette smoke for 3 months) and (**c**) in the S-Em model (*n* = 6 mice for the control and *n* = 5 mice for the cohort treated with cigarette smoke for 8 months).

Given the supportive role of microglia in microvascular remodeling, we next assessed microglia density in the same regions as in the microvascular measurements. We performed immunofluorescence staining of the microglia/macrophage-specific marker protein Ionized calcium-binding adaptor molecule 1 (Iba1) in combination with DNA staining to determine the proportion of Iba1^+^ cells in the regions of interest. The cerebellum was excluded from these analyses due to its structural heterogeneity, with certain regions containing very high cell densities^34^ that prevent accurate and reproducible DAPI-positive cell counting. Although subtle trends in microglial density appeared to correlate with changes in microvascular density in some regions, none of these changes proved to be statistically significant (Fig. 3a–c, Supplementary Figure S3). Since the hippocampus showed the most profound and significant changes in microvascular remodeling, we performed additional nearest neighbor distance (NND) analyses of microglia in this region. As with microglia density, no significant change was observed in microglia distribution (Supplementary Figure S4). Overall, these results suggest that our analyses captured the already remodeled states of the brain microvasculature, rather than ongoing remodeling processes, or that the remodeling was too subtle falling below the detection limit at these time points.

Results of quantitative immunohistochemical analyses of the hypoxia-inducible transcription factors HIF1a and HIF2a, markers for acute and chronic hypoxia, respectively^35^, also suggested a largely adapted microenvironment, as no significant increase in these markers was observed in the treatment groups (Supplementary Figure S5 and Supplementary Table 2).

### 3D-imaging of cleared 250 μm tissue sections supports and extends the view of adaptive brain microenvironment remodeling in S-PH and S-Em mice

**Fig. 4:**
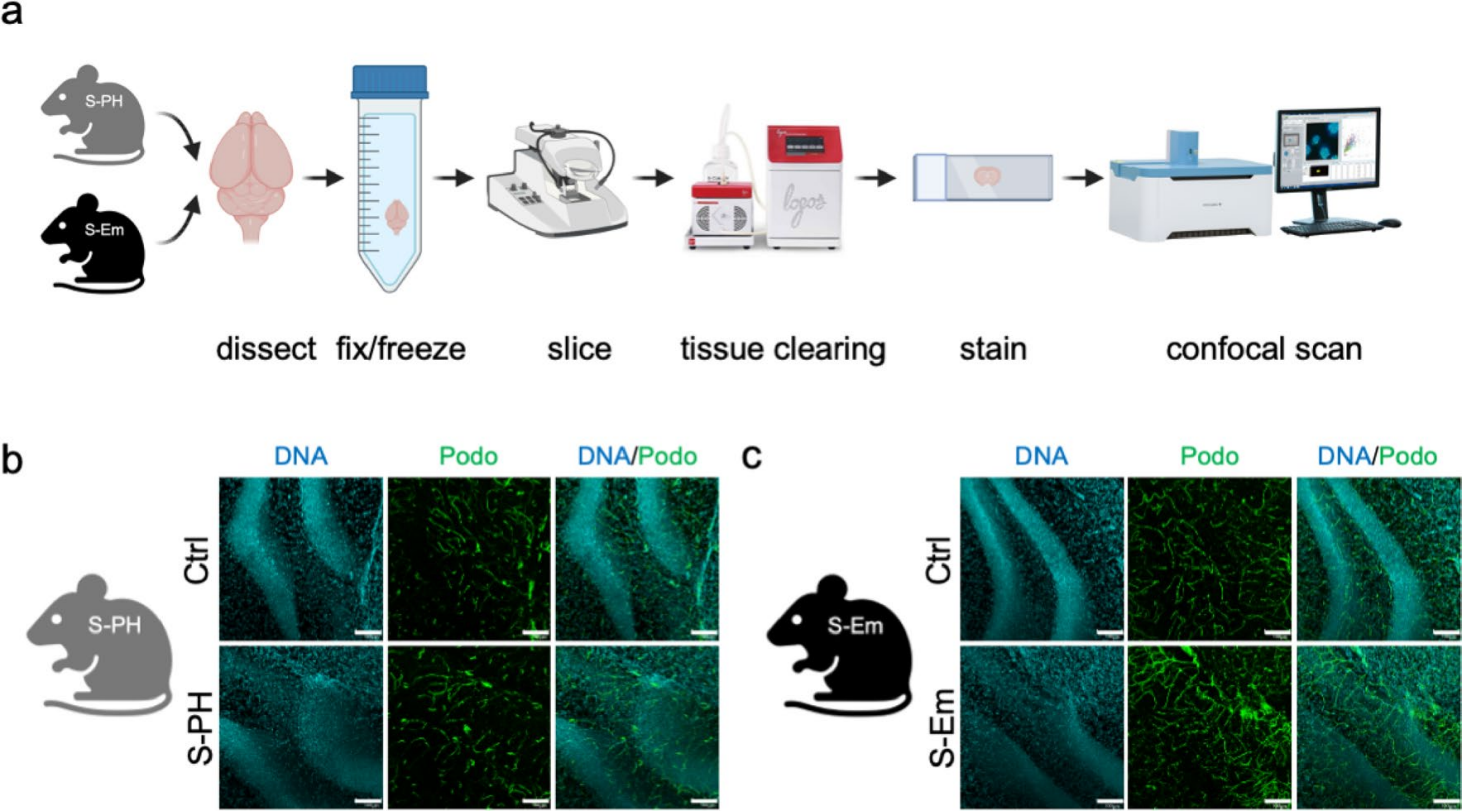
Microvasculature staining in 250 μm thick sections in the mouse models of smoke exposure. (**a**) Workflow scheme of immunofluorescence analyses of 250 μm tissue sections. Z-projections of serial confocal images of endothelial marker podocalyxin staining in the (**b**) S-PH and (**c**) S-Em model. Representative hippocampal ROIs with DAPI (DNA) staining and podocalyxin staining (Podo) are shown. Scale bars indicate 100 μm.

To validate and extend the observations on the brain microvasculature and microglia status obtained by quantitative immunofluorescence analyses of 5 μm thin FFPE tissue sections, we performed 3D-imaging analyses in the smoke exposure models. Fixed mouse brain hemispheres were cut into 250 μm thick sections and subjected to tissue clearing and subsequent podocalyxin and Iba1 immunofluorescence staining (Fig. 4a). While our focus on the hippocampus - chosen for its pronounced changes in microvascular density - constrain the generalizability of our findings, 3D imaging (Fig. 4b, c) still confirms that this region exhibits the greatest increases in microvascular density in the S‑Em model (mean %area is 3.06% in control vs. 3.50% in S-Em) and a slightly decreased microvascular density in the S-PH model (mean %area is 2.36% in the control vs. 2.14% in S-PH), supporting the quantitative 2D analyses. Microglia stained with Iba1 displayed morphological variations (Supplementary Figure S6 and Supplementary Video S1), and the detailed recording of cell morphology provides a basis for further analysis. However, this will require significantly larger sample sizes.

### Fibrinogen staining indicates that no cerebrovascular leakage occurs at the analysis time points

**Fig. 5:**
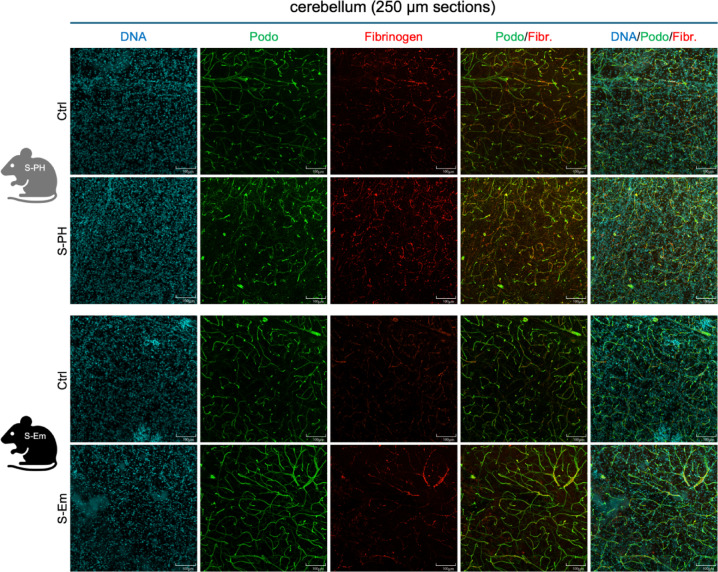
Vascular leakage marker fibrinogen and endothelial marker podocalyxin co-staining. Z-projections of serial confocal images of endothelial marker podocalyxin/fibrinogen immunofluorescence co-staining in the S-PH and S-Em model. Representative cerebellar ROIs are shown. Scale bars indicate 100 μm.

To evaluate cerebrovascular leakage, we performed immunofluorescence staining of fibrinogen, a marker for vascular leakage, alongside podocalyxin. Subsequent 2D/3D image analysis corroborated the notion that the time points of our analyses did not overlap with intense remodeling of the brain’s microvasculature which would be accompanied by increased leakage due to impaired microvascular integrity. Analyses of 5 μm tissue sections from CMH, S-PH and S-Em mice, showed no fibrinogen signal outside blood vessels (Supplementary Figure S7). Three-dimensional immunofluorescence images of 250 μm tissue sections from S-PH and S-Em mice reinforced the finding of solely intravascular fibrinogen localization, as shown in Z-projections (Fig. 5) as well as in 3D rotating views and Z-stack image series (Supplementary Videos S2 and S3) of exemplary cerebellar regions that display high microvascular density. These results align with our microvasculature and microglia analyses, indicating that our study time points did not capture significant acute microvascular remodeling, which would typically be associated with cerebrovascular leakage. Our observations might reflect post-remodeling statuses which may not be irreversible, given the homeostatic capacity of the brain microvasculature, or they could indicate a subtle, continuous microvascular remodeling process that evades detection in our temporal snapshots due to the limited sensitivity of our methodology.

### Fibrinogen and MMP-9 levels display different systemic and brain organotypic alterations in the CMH and S-PH, S-EM models

**Fig. 6:**
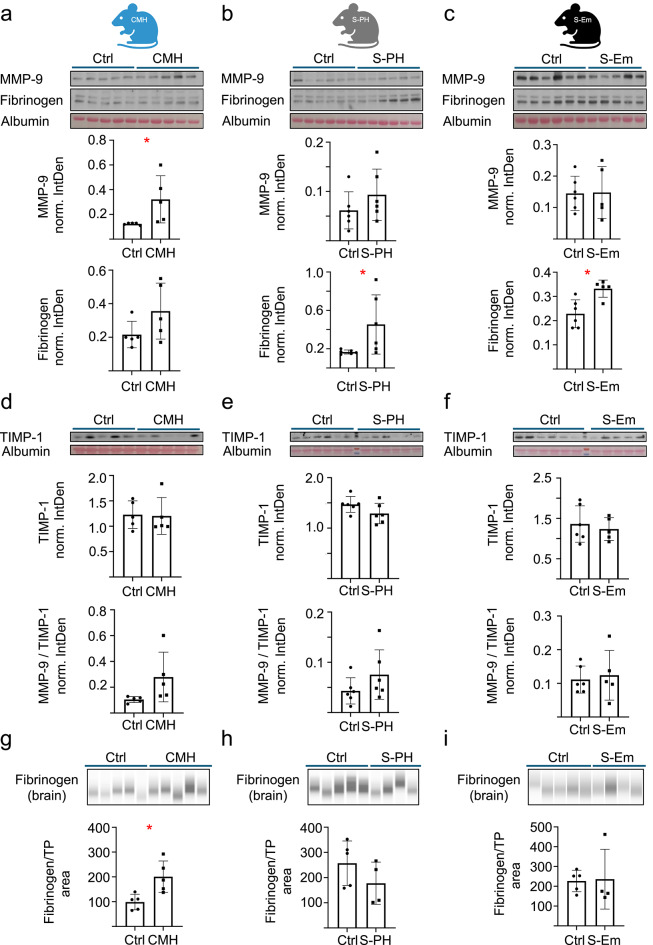
Immunoblot analyses of fibrinogen and MMP-9. Western blot and quantitative evaluation of fibrinogen and MMP-9 protein levels of blood plasma samples (**a**) in the CMH model (*n* = 5 mice for both control and for the cohort treated with 10% O_2_ for 28 days), (**b**) in the S-PH model (*n* = 6 mice for both the control and for the cohort treated with cigarette smoke for 3 months) and (**c**) in the S-Em model (*n* = 6 mice for the control and *n* = 5 for the cohort treated with cigarette smoke for 8 months). The integrated densities of the immunodetections of fibrinogen and MMP-9 were normalized to the albumin densities measured after Ponceau staining and are given as norm. IntDen. (**d**–**f**) Western blot and quantitative evaluation of TIMP-1 protein levels of the same blood plasma samples as in (a-c). In addition, the MMP-9/TIMP-1 norm. IntDen. ratios are also shown. Capillary-based protein immunoassay detection of fibrinogen protein levels of brain FFPE tissue samples (**g**) in the CMH model (*n* = 5 mice for both control and for the cohort treated with 10% O_2_ for 28 days), (**h**) in the S-PH model (*n* = 5 mice for the control and *n* = 4 mice for the cohort treated with cigarette smoke for 3 months) and (**i**) in the S-Em model (*n* = 5 mice for the control and *n* = 4 for the cohort treated with cigarette smoke for 8 months). Fibrinogen levels were normalized to the total protein (TP) level using the JESS capillary-based instrument and are shown as Fibrinogen/TP area. Unpaired t-tests *p* < 0.05 are marked with a red asterisk.

Fibrinogen is not only a marker for cerebrovascular leakage but has also been described as a driver of endothelial cell permeability^17^. The mechanism involves downregulation of vascular endothelial cadherin and activation of matrix metalloproteinase 9 (MMP-9)^19^. To investigate the systemic changes in fibrinogen and MMP-9 levels associated with hypoxia and smoke exposure, we performed immunoblot analyses of blood plasma samples from CMH, S-PH and S-Em mice. All samples were analyzed in two separate runs, with the order of loading randomized in the second run. Our analyses showed increased MMP-9 levels in CMH mice and no significant MMP-9 changes and trends in S-PH and S-Em mice, whereas fibrinogen levels showed an increase in S-PH and S-Em mice (Fig. 6a–c and Supplementary Figure S8). Importantly, circulating MMP-9 in the blood is present largely as inactive pro-MMP-9 zymogen, and upon activation can be still blocked by circulating tissue inhibitor of metalloproteinases 1 (TIMP-1)^36^. The immunoblot analyses detected a prominent signal at > 95 kDa (Supplementary Figure S8) and no signal between 72 kDa and 95 kDa. This result indicates that the vast majority of MMP-9 is present in its zymogen form, as pro-MMP-9 and MMP-9 are 92 kDa and 82 kDa respectively without glycosylation. To investigate further if potential zymogen activation could lead to markedly different activities in the blood of control and treated mice, we measured TIMP-1 levels in the plasma samples. The results showed no significant changes upon any treatment (Fig. 6d–f, Supplementary Figure S8). Moreover, when we calculated MMP-9/TIMP-1 level ratios, there was no significant increase in the CMH group in contrast to MMP-9 only. These results suggest that a possible involvement of the fibrinogen-MMP-9/TIMP-1axis in microvascular remodeling would require site-specific activation in the brain. To assess the organotypic changes, we then prepared protein extracts from brain FFPE samples adjacent to the cortex, hippocampus and brainstem containing regions analyzed in immunofluorescence experiments. They were subjected to capillary immunoblotting and, due to technical limitations, only fibrinogen analysis. While significantly increased levels were detected in CMH mice, a decreasing tendency was observed in S-PH mice and no change in S-Em mice. Overall, systemic increase in fibrinogen levels in the smoke exposure model did not lead to detectable fibrinogen accumulation in the brain.

## Discussion

In this study, we used mouse models to investigate the co-evolution of chronic lung diseases and changes in the brain microenvironment. A common consequence of lung dysfunction is impaired gas exchange in the alveoli. This leads to hypoxemia, characterized by reduced oxygen level in the blood and ultimately resulting in chronic hypoxia, i.e. decreased O_2_ concentrations. This condition was simulated in the CMH experiments. Importantly, the brain is highly sensitive to changes in O_2_ levels^37^, which correlates with pronounced microvascular remodeling compared to other organs^12^. It is also crucial to note that these changes are accompanied by a systemic, organ-specific metabolic shift; in particular with chronic hypoxia promoting fatty acid uptake and oxidation in the brain^38^. In addition to the CMH model, the S-PH and S-Em mice are exposed to various effects induced by tobacco smoke, including but not limited to increased concentrations of reactive oxygen and nitrogen species (ROS/RNS) and inflammatory signaling, that lead to PH and emphysema development^31^, and also cause microvascular remodeling in the brain. Overall, our results indicate that microvascular loss predominates in S-PH. While Fgf10-specific mechanisms have been extensively studied in the lung^39^, their potential brain-specific functions in S-PH mice still need to be investigated. The differently triggered adaptive mechanisms in the brain in the CMH and S-Em models appear to lead to increased microvascular density. These findings prompt us to consider which changes caused by the different stresses underlie the respective phenotypes. Although there is overlap in the inflammatory responses, certain transcriptional signatures are more characteristic of chronic smoke-exposed mouse models of lung injury than of hypoxia models. For example, Ccl6, Ccl25, Vegfb and Fgf10 are more specifically associated with tissue damage and remodeling in chronic cigarette smoke exposure than in chronic hypoxia-induced PH of comparable severity^31^. These signatures point to a specific inflammation‑driven mechanism in the S‑PH/S‑Em models, which causes microvascular remodeling in the brain in a different way than systemic hypoxia in the CMH model. For the smoke exposure scenario, we propose that the observed changes reflect time-dependent smoke-induced inflammation that initially leads to a loss of microvessels - particularly in the hippocampus - followed by a secondary adaptive response to the resulting tissue hypoxia and persistent smoke-specific inflammatory signals. Our study highlights distinct patterns of local microvascular density changes across these models, suggesting that specific causative agents and their time‑point‑specific effects govern brain microvascular homeostasis and could explain the differing temporal dynamics of remodeling.

Based on recent research in mouse models, the brain exhibits significant regional heterogeneity in microvascularization. The hippocampus stands out as having lower blood flow, lower oxygen saturation, and higher susceptibility to stress (e.g. inflammatory, oxidative, hypoxic and metabolic) compared to the neocortex, while the brainstem and cerebellum tend to be better perfused and thus more stress‑tolerant^40–43^. Moreover, reduced angiogenic capacity in the hippocampus compared to the cortex was identified as an additional cause of regional vulnerability^44^. It is also important to note that COPD and cigarette smoke exposure have clearly region‑biased effects in the human brain and in mouse models, with the hippocampus emerging as the most consistently affected structure, while the cortex, brain stem, and cerebellum show milder or less well-documented changes, for which we refer to a detailed recent review article^45^. Our observations, showing the most significant changes in the hippocampus, are largely consistent with these findings. While our analyses did not directly detect ongoing microvascular remodeling, the decreased vessel densities in the S-PH model (after 3 months of tobacco-smoke exposure) and increased densities in the S-Em model (after 8 months of tobacco-smoke exposure) strongly suggest that region-specific microvascular remodeling must take place. Increased vessel density combined with impaired BBB integrity may contribute to various neuropathological settings. A recent study demonstrated that tobacco-smoke-induced COPD with or without additional pro-inflammatory lipopolysaccharide treatment is associated with reduced levels of BBB marker proteins^46^, as an indicator of impaired BBB function. Of note, the animal model used in the study is more similar to our S-PH model (72-days vs. 3-months exposure), in which we observed lower vessel density. It remains to be determined whether decreased BBB protein levels are merely a result of decreased vessel density or represent ongoing BBB dysfunction. While the mechanisms governing the spread of metastasis to the brain remain partially elusive, it is noteworthy that the role of the BBB in promoting both pre-metastatic and metastatic microenvironments is increasingly understood^47–50^. Although not brain-specific, endothelial dysfunction is a hallmark of COPD^51^, and the role of hypoxia in enhancing neutrophil-mediated endothelial damage is well documented^52^. Overall, our observations on the changes in brain microvascular remodeling contribute to the study of neuropathological comorbidities of chronic lung diseases caused by smoke exposure or hypoxia. However, we would like to emphasize that the connection between our findings and metastases is currently only a hypothesis and not a proven mechanism.

The well-established, yet incompletely understood role of microglia - brain resident innate immune cells - in malignancies and other central nervous system pathologies^53–58^, and in particular their function in microvascular remodeling^12–15,59^ and their response to nicotine during metastasis^60^, prompted us to investigate microglia in the CMH, S-PH and S-Em model systems. Notably, in the context of smoke-induced pulmonary hypertension in mice, we have already identified myeloid cell-specific mechanisms that critically contribute to a pro-inflammatory environment and pulmonary vascular remodeling^61^. The lack of detectable significant changes in microglia densities or distribution with our methods suggest that a higher temporal resolution and/or higher sensitivity is needed. It is interesting to note here that although adult microglia are more strongly associated with capillaries than their newborn and aged counterparts, they have a more stable morphology and demonstrate rapid response and clearance in acute, focal laser-induced injury^62^. To capture possible smoke-exposure-related specific changes, we first applied a 3D-imaging approach on cleared 250 μm thick tissue samples of the smoke exposure model. The results of these experiments align well with the observations from conventional 5 μm thick FFPE sections. Overall, our data and the studies mentioned above imply that the involvement of microglia in microvascular remodeling may be temporary or functional rather than based on their numbers and general distribution. Thus, our aim is to collect a larger quantity of high-quality microglia 3D structures for advanced structural analysis and modeling alongside molecular characterization of isolated microglia cell populations^63^.

To initially assess the role of a potential contributor to changes in microvascular and microglia states we also investigated fibrinogen leakage in brain tissue sections. Our 2D- and 3D-imaging results indicate that fibrinogen is largely intravascular at the time of analyses, without detectable extravascular fraction in the brain parenchyma, which would be a sign of cerebrovascular leakage. While no fibrinogen leakage was detected in our immunofluorescence analyses in CMH, S-PH and S-Em mice, the staining was more pronounced compared to controls. This invites to speculate on the possible contribution of fibrinogen to blood-brain barrier disruption or microglia activation. Considering the known systemic and organotypic changes in fibrinogen levels, its outlined brain-specific roles and our observations in immunofluorescence analyses, we further investigated fibrinogen and MMP-9 levels by immunoblotting. The increased systemic (plasma) MMP-9 levels in the CMH mice did not coincide with increased plasma fibrinogen levels. The increasing tendency without significant changes in plasma fibrinogen levels was consistent with previous findings^30^. Interestingly, we found a brain organotypic increase in fibrinogen levels in CMH mice at the same time. This observation, as well as the increased vessel density are in agreement with a model proposed by Halder and Milner^14^, in which hypoxia-induced endothelial proliferation induces transient fibrinogen deposition within the walls of angiogenic blood vessels, but no vascular leakage. Whether and how the increased MMP-9 plasma levels together with the locally increased fibrinogen levels could contribute to microvascular remodeling in the brain remains to be investigated further. In contrast to the CMH mice, plasma MMP-9 levels and brain fibrinogen levels were unchanged in the S-PH and S-Em mice, and an increase in plasma fibrinogen levels was observed. This parallels COPD^28,29^, and may be a trigger for adaptation to smoke exposure, with the S-PH mice and S-Em mice possibly reflecting different stages. However, the picture is still controversial and incomplete in some respects, suggesting that further research is needed to gain a comprehensive understanding of the topic. The rapidly developing molecular maps of the vasculature, such as the organotypic atlas of vascular heterogeneity^64^, will provide very useful resources for the assessment, prediction and evaluation of specific, local effects of fibrinogen and other plasma factors. Additionally, exploring the relationship between systemic inflammation in COPD and fibrinogen-mediated changes in the brain microvasculature could provide valuable insights into the mechanisms underlying their potential role in modulating metastatic susceptibility. The microvascular changes observed may contribute to conditions compatible with a multifaceted pre-metastatic niche. Reduced capillary density and loss of endothelial integrity may create a mechanically “high-shear” microenvironment with low flow, facilitating the extravasation of tumor cells. At the same time, the dysfunctional endothelium and damaged blood-brain barrier have been associated with the release of proinflammatory cytokines (e.g. IL-6, TNF-α, VEGF) and chemokines (e.g. CXCL1, CCL2, CXCL8), forming gradients that attract bone marrow-derived myeloid cells and potentially render the niche tumor-supportive. Elevated fibrinogen, which can leak from damaged vessels during active microvascular remodeling, can polymerize into fibrin scaffolds and form integrin binding sites that may further support the adhesion and growth of tumor cells. Together, the intertwined mechanical and biochemical changes surrounding the damaged microvascular system, in conjunction with circulating tumor-derived factors, may create a pre-metastatic microenvironment permissive for metastatic seeding, although the present study does not directly assess niche formation or metastatic progression.

Despite the promising findings, our study has some limitations that should be considered. The use of animal models provides valuable insights into the mechanisms underlying COPD and the co-evolution of brain-related changes that potentially facilitate comorbidities such as brain metastasis. However, the small sample size, and limited diversity of animals may restrict the generalizability of the results. Additionally, the selective choice of brain areas studied may not fully capture the complexity of microvascular remodeling across the entire brain. While trends in hippocampal microvascularization, which is highly sensitive to hypoxia, or in MMP-9 and fibrinogen levels are evident, the use of immunofluorescence and immunoblot techniques limits absolute quantification, and the small cohort size restricts robust statistical analyses. Consequently, our conclusions may not apply to untested brain regions, and species-specific or age-specific variations cannot be excluded. Moreover, it would be beneficial to determine the evolution rate and stability of microvascular statuses and combine our model systems with mouse lung cancer models. Nevertheless, this study lays the groundwork for further investigations and provides novel insights into how chronic lung diseases, such as lung emphysema, and changes in the brain microenvironment co-evolve and may contribute to a supportive setting for neuropathological comorbidities. Future studies should longitudinally track microvascular remodeling dynamics to map the temporal sequence of capillary loss and compensatory sprouting in the smoke-exposure model. Furthermore, the impact of the observed microvascular remodeling should be investigated in clinically relevant lung‑cancer brain‑metastasis models by quantifying metastatic lesion formation in CMH, S-PH and S-Em mice. Finally, additional target‑gene analyses should be performed e.g. via bulk and single‑cell RNA‑seq of isolated endothelial cells, pericytes, and microglia from affected brain regions to uncover cell‑type‑specific transcriptional programs that drive the observed vascular alterations.

In conclusion, this pioneering comparative exploration of key components of the mouse brain microenvironment in several mouse model settings offers a valuable foundation for future research. By examining distinct patterns of microvascular remodeling, we underscore the complex interplay between systemic-hypoxia- or tobacco-smoke-induced stress, and time-specific changes in microvascular dynamics. This study provides initial evidence for associated lung-brain alterations in the context of chronic lung diseases. The relevance of these findings to specific neuropathological conditions, including lung cancer brain metastases, warrants further investigation in future studies e.g. with lung cancer models. Our findings pave the way for advancing our understanding of how chronic lung diseases are related to the progression of brain-related comorbidities.

## Materials and methods

### Animal experiments

Wildtype C57BL/6J mice, 10–12 weeks old, were obtained from Charles River Laboratories, Sulzfeld, Germany. Animals were housed under controlled conditions at 22 ± 1 °C and ~ 50% humidity with a 12 h light/dark cycle and food and water supply ad libitum. Animals were monitored daily for general health status and signs of distress. Animals were to be removed from the study if they developed severe symptoms (e.g. breathing difficulties). Humane endpoints were predefined by applying established experimental models described in the two paragraphs below. Animals were randomly allocated to control and treated (tobacco-smoke exposed or hypoxia) groups. All experiments were approved by the governmental ethics committee for animal welfare of the Regierungspräsidium Gießen (https://rp-giessen.hessen.de), Germany (ethical approval codes G25/2019 and G41/2019). We confirm that all methods were carried out in accordance with the relevant guidelines and regulations and all methods are reported in accordance with ARRIVE guidelines (https://arriveguidelines.org). Both male and female mice were used in all experimental groups and gender was not used as an exclusion criterion.

### Chronic mild hypoxia (CMH) mouse model

To induce chronic mild hypoxia (CMH) in the brain, 10–12 weeks old male and female C57BL6/J wildtype mice were exposed to normobaric chronic hypoxia (10% O_2_) for 28 days as described previously^65,66^. Briefly, mice were kept in a ventilated chamber (Biospherix Ltd., Lacona, NY, USA) in normobaric hypoxia (fraction of inspired O_2_ [FiO_2_], 0.10). The control mice (Ctrl) were kept under comparable normoxic conditions (FiO_2_, 0.21). The chambers were only opened for a period of less than 10 min every day as required, to prevent reoxygenation.

### Tobacco-smoke exposure mouse model

Two stages of a tobacco-smoke exposure mouse model were used as described previously^31,39,67,68^. In 10–12 weeks old male and female C57BL6/J wildtype mice the 3-month exposure leads to pulmonary hypertension without emphysema (S-PH), while the 8-month exposure causes emphysema in addition (S-Em). Briefly, male and female wild-type C57BL6/J mice were exposed to mainstream smoke of 3R4F cigarettes (Lexington, KY, USA) at 200 mg particulate matter/m^3^ for 6 h/day, 5 days/week for 3 months or 8 months. Cigarette smoke was produced by a semi-automatic generator (Burghart GmbH, Wedel, Germany) as previously described^31,67,69^. Age-matched controls (Ctrl, 10–12 weeks plus 3 months old for S-PH, 10–12 weeks plus 8 months old for S-PH) were kept under identical conditions without tobacco-smoke exposure.

### Animal preparation, in vivo hemodynamics, lung function

Measurements of in vivo lung function and hemodynamic measurements were performed as previously described^67–69^, with slight modifications. Briefly, anesthesia was induced by putting a mouse in a chamber with 3% isoflurane in 100% O_2_. The mouse was then placed on a thermoregulation plate with constant anesthesia supply via a nose-only mask. Electrocardiogram (ECG) electrodes and a rectal thermometer (Indus Instruments, Houston, TX, USA) were placed for monitoring the heart beating frequency and body temperature. After tracheotomy, the animals were intubated with 18 G metal tubus (SCIREQ Scientific Respiratory Equipment Inc., Montreal, QC, Canada). The trachea was fixed with a thread and ventilated, using a FlexiVent system, equipped with an FX2 module (SCIREQ Scientific Respiratory Equipment Inc., Montreal, QC, Canada), at a frequency of 150 breaths/min and a tidal volume of 5 ml/kg.

Lung function tests were performed as previously described^67,70^, using the FlexiVent predetermined script at positive end-expiratory pressure (PEEP) of 3 cmH_2_O, with a consistent perturbation order, following the manufacturer’s recommendations. Briefly, prior to the lung function measurement, deep inflation was performed as a recruitment manoeuvre. The deep inflation consisted of inflating the lung with air pressure from 3 to 40 cmH_2_O over 3 s and then holding at 40 cmH_2_O for additional 3 s. That way, closed lung areas were recruited, and lung volume history was standardized^71,72^. Dynamic compliance was measured using a single frequency forced oscillation (SnapShot-150) perturbation^71^. Static compliance measurement was obtained from a respiratory pressure-volume (P-V) loop^71^. The P-V maneuver included stepwise lung inflation through 8 steps of increasing pressure from 3 to 40 cmH_2_O with a 1-second hold at each step. It was then followed by stepwise deflation in a similar manner back to PEEP pressure of 3 cmH_2_O. The whole maneuver lasted 16 s. The P-V loop was created by recording volume changes and plotting it with pressure values at each holding step^71^. The results were presented as an average of at least three repeated measurements with the coefficient of determination (COD) above 0.95.

Hemodynamic measurements were performed as previously described^67–70^. Briefly, for right ventricular systolic pressure (RVSP) measurements, the jugular vein was catheterized by a micro-tip catheter (SPR 671 REF 8406719; Millar Instruments Inc., Houston, TX, USA) that was then forwarded into the right ventricle. Mean arterial and left ventricular systolic pressures (mAP and LVSP, respectively) were measured by a catheter inserted through the carotid artery into the aorta and then in the left ventricle. The animals were afterwards sacrificed by exsanguination through the carotid artery. Measurements were recorded and analyzed using the PowerLab system and LabChart 7.0 software (AD Instruments GmbH, Spechbach, Germany). To measure right ventricular hypertrophy, the right ventricle (RV) was separated from the left ventricle and septum (LV + S), and their masses were weighed. To determine hematocrit levels, hematocrit capillaries were filled with the previously collected blood and sealed at the lower end. The capillaries were centrifuged at 15,570 rpm for 5 min at room temperature. The hematocrit value was then obtained by reading it off the hematocrit scale.

### Immunohistochemistry and immunofluorescence imaging of standard FFPE sections

Right ventricular-perfused brains were cut through the frontal axis at the bregma (0) point, fixed in 4% paraformaldehyde for 24 h and embedded in paraffin (FFPE). FFPE tissue sections were cut using a Leica SM 200R microtome (Leica Microsystems GmbH, Wetzlar, Germany) with 5 μm thickness and used for immunohistochemistry (IHC), and immunofluorescence (IF) staining. To minimize differences in staining and image quality, paired batches of control and treatment slides (Ctrl/CMH, Ctrl/S-PH, Ctrl/S-Em) were processed simultaneously.

IHC was performed essentially as described previously^73^. Briefly, after deparaffinization and rehydration of the sections, antigen retrieval was carried out using citrate buffer (pH 6.0). Sections were then incubated with 0.6% H₂O₂ for 30 min to block endogenous peroxidase activity and blocked with 20% normal goat serum in PBST (PBS containing 0.1% Tween-20). After removal the blocking buffer, the sections were incubated with primary antibodies (diluted in 10% normal goat serum (NGS) in PBST) for 2 h at room temperature (RT), followed by PBST washes. Primary antibody information and dilutions are listed in Table 1. Subsequently, sections were incubated with a biotinylated swine anti-rabbit secondary antibody (diluted 1:200 in 10% NGS in PBST) for 1 h at RT. After additional washes in PBST, sections were treated with ABC reagent for 45 min. The chromogen reaction was developed by diaminobenzidine (DAB) substrate for up to 5 min, and the reaction was stopped by transferring sections to PBS. Sections were counterstained with hematoxylin for 2 min and then dehydrated. Finally, sections were mounted using Cytoseal XYL and imaged on an Axio Scan.Z1 slide scanner (Carl Zeiss Microscopy GmbH, Jena, Germany) using a 20x objective and the ZEN 2.3 software for acquisition.

For IF staining, sections were initially deparaffinized and rehydrated. The sections were incubated with MaxBlock (MaxVision Biosciences, MB-L) Reagent A for 10 min to reduce tissue autofluorescence, followed by immersion in 60% ethanol for 1 min. Subsequently, the sections were washed (all washing steps were 3 × 5 min unless otherwise stated) with distilled water and PBST (PBS containing 0.001% Triton X-100). Post-fixation was carried out using cold 2% paraformaldehyde (PFA) for 5 min. Antigen retrieval was achieved using either citrate buffer (pH 6) or Tris-EDTA (TE) buffer (pH 9), depending on primary antibody specifications detailed in Table 1, followed by washes in PBST. The sections were permeabilized in PBS containing 0.5% Triton X-100 for 20 min followed by blocking with 2% bovine serum albumin (BSA) in PBS with 0.5% Triton X-100 for 30 min. Primary antibodies were applied overnight at 4 °C in an antibody dilution buffer (1% BSA in PBS with 0.25% Triton X-100) followed by washes in PBST. Primary antibody information and dilutions are listed in Table 1. The next day, sections were incubated with the appropriate fluorophore-conjugated secondary antibodies (diluted 1:500 in antibody dilution buffer) for 2 h at RT followed by washes in PBST. For post-detection, sections were incubated with the MaxBlock Reagent B for 5 min and rinsed in distilled water. Tissue sections were counterstained with DAPI (5 mg/ml stock diluted 1:5000 in PBS) for 10 min, followed by washes in PBS (2 × 5 min) and in distilled water (1 × 5 min) prior to mounting with Dako (S3023) fluorescence mounting media. Sections were imaged on an Axio Scan.Z1 slide scanner (Carl Zeiss Microscopy GmbH, Jena, Germany) using a 20x objective and the ZEN 2.3 software for acquisition.

For the podocalyxin-fibrinogen co-staining, sections were first incubated with an anti-podocalyxin primary antibody followed by a secondary antibody. Afterward, the sections were incubated with an anti-fibrinogen antibody overnight at 4 °C and then with the corresponding secondary antibodies (antibody information and dilutions are listed in Table 1). Imaging was performed on a CQ1 spinning disc confocal microscope (Yokogawa Life Science, Tokyo, Japan; Cenibra GmbH, Bramsche, Germany) using 405/488/561 nm lasers and a 20x objective.

The selection of coronal sections and regions of interest (ROIs) for image analyses is described in detail in the section Image analysis.

**Table 1:** Antibodies and conditions used in IHC and IF staining.

Antibodies	Host	Type	Clonality	Supplier	Cat. number	Antigen retrieval	Dilution
IHC							
anti-HIF1α	Rabbit	Primary	Polyclonal	Cayman Chemical	10006421	Citrate buffer pH 6	1:500
anti-HIF2α	Rabbit	Primary	Polyclonal	Novus Bio	NB 100–122	Citrate buffer pH 6	1:200
anti-Rabbit Biotin	Swine	Secondary	Polyclonal	Dako	E0353		
IF							
anti-Iba1	Rabbit	Primary	Polyclonal	Wako Chemicals	019-19741	TE buffer pH 9	1:1000
anti-Podocalyxin	Goat	Primary	Polyclonal	R&D Systems	AF1556	Citrate buffer pH 6	1:100
anti-Fibrinogen	Rabbit	Primary	Polyclonal	Abcam	ab34269	Citrate buffer pH 6	1:200
anti-Goat Alexa Fluor 488	Donkey	Secondary	Polyclonal	Invitrogen	A-11055		
anti-Goat Alexa Fluor 568	Donkey	Secondary	Polyclonal	Invitrogen	A-11057		
anti-Rabbit Alexa Fluor 568	Donkey	Secondary	Polyclonal	Invitrogen	A-10042		

### Tissue clearing and immunofluorescence imaging of 250 μm thick fresh frozen sections

The right ventricular-perfused brains were fixed in 4% paraformaldehyde for 24 h and sectioned using a Leica VT 1200 S vibratome (Leica Microsystems GmbH, Wetzlar, Germany) at a thickness of 250 μm. Electrophoretic tissue clearing was performed using the X-CLARITY system (Logos Biosystems, Villeneuve d’Ascq, France; BioCat GmbH, Heidelberg, Germany) with the following settings: 70 V, 1.2 A, 37 °C for 2 h. To further reduce autofluorescence, the cleared brain sections were bleached with 4.5% (w/v) H_2_O_2_ and 20 mM NaOH in PBS for 90 min (2 × 45 min), followed by washing in PBS for 1 h (4 × 15 min) on a shaker. The sections were then permeabilized with 0.5% Triton X-100 in PBS for 2.5 h and blocked with 3% BSA in 0.1% Triton X-100 PBS for 4 h on a shaker. An anti-podocalyxin primary antibody (1:100 in PBS containing 1.5% BSA and 0.1% Triton X-100) was applied for 2 days at RT on a shaker. After washing with PBST (PBS containing 0.1%Triton X-100) for 1 h (4 × 15 min), sections were incubated with donkey anti-goat Alexa Fluor 488 secondary antibody for 5 h at RT on a shaker. Following washing steps, sections were incubated overnight at RT with either anti-Iba1 (diluted 1:400 in 1.5% BSA in PBST) or anti-fibrinogen (diluted 1:200 in 1.5% BSA in PBST) primary antibodies, followed by incubation with donkey anti-rabbit Alexa Fluor 568 secondary antibodies for 5 h on a shaker. After further washes in PBST (4 × 15 min), sections were counterstained with DAPI (5 mg/ml stock diluted 1:2500 in PBS) for 10 min, washed in PBS (3 × 15 min) and in distilled water (15 min). Finally, sections were incubated with X-CLARITY™ Mounting Solution for 1 h (2 × 30 min) at RT on a shaker and mounted.

Imaging was performed on a CQ1 spinning disc confocal microscope (Yokogawa Life Science, Tokyo, Japan; Cenibra GmbH, Bramsche, Germany) using 405/488/561 nm lasers and 20x, or 40x objective with z-stack imaging (250 μm range, 2 μm step size, 100% excitation power, 500 ms exposure per channel).

### Image analysis

The Qupath software (v 0.3.2) was used to analyze HIF1α and HIF2α staining results. For each sample, three regions of interest (ROIs) were selected from the hippocampus to cover all major subregions, including CA3/CA4, CA2 and CA1, while four ROIs were selected from cerebral cortex, with two from the medial cortex and two from the lateral cortex. The position of ROIs is indicated on the graphical illustration in Supplementary Table 1. The ‘Positive cell detection’ function was applied to identify DAB+ cells. Intensity threshold parameters were set as follows: Threshold 1+: 0.2, Threshold 2+: 0.3, Threshold 3+: 0.4. Threshold values were defined based on negative control sections (without primary antibody) and visually verified to distinguish DAB-positive cells from background staining. After fixing the threshold values, identical threshold settings were applied to all samples. To analyze the podocalyxin and Iba1 staining, ROIs were defined and groups of them were categorized by major anatomical regions according to the Allen Mouse Brain Atlas (Coronal Atlas, Mouse, P56. 2011 release at https://mouse.brain-map.org/static/atlas) as follows: 3 ROIs in the cortex (in the region according to images 72 to 75), 6 ROIs in the hippocampus (in the region according to images 72 to 75), 5 ROIs in the brain stem (in the region according to image 72 to 75), and 4 ROIs in the cerebellum (in the region according to images 115 to 120). The position of ROIs is indicated on the graphical illustrations in Fig. 2a and Supplementary Tables S1 and S2. Fiji software (ImageJ2 Version 2.14.0/1.54f) was used to analyze podocalyxin staining.

Thick sections with podocalyxin/fibrinogen co-staining were used for qualitative assessment of fibrinogen localization (microvascular leakage) with higher sensitivity compared to thin section analyses. To assess changes in microvascular density and possible changes in microglia morphology on thick samples with podocalyxin/Iba1 co-staining, the hippocampal region was selected, where the most severe microvascular changes were detected in extensive quantitative analyses of standard FFPE sections and where microglia changes were therefore suspected. Confocal imaging optical sections were used as input to quantify podocalyxin staining in the thick sections shown in Fig. 4. Mid sections of 2 μm width were selected from a 100 μm wide region (every 5th section, totaling 11 sections per sample (S-PH/Ctrl and S-Em/Ctrl)) of the 250 μm thick sections and the mean %area values of the vessel densities were calculated for the individual samples. Images were opened in Fiji and set scale accordingly. Images were then converted to 8-bit, and the Otsu automated threshold function was applied. Subsequently the vessel density plugin https://imagej.net/plugins/vessel-analysis was run and the results were recorded.

Iba1^+^ cells were manually counted with the QuPath software. White circles were used to mark each Iba1^+^ cell, and images with marked cells were exported to determine the distribution of Iba1^+^ cells.

Nuclei were counted using the following script in QuPath: “setPixelSizeMicrons(0.227000, 0.227000); createSelectAllObject(true); runPlugin(‘qupath.imagej.detect.cells.WatershedCellDetection’, ‘{“detectionImage”:“Blue”, “requestedPixelSizeMicrons”:0.227, “backgroundRadiusMicrons”:4.0, “medianRadiusMicrons”:0.0, “sigmaMicrons”:3,“minAreaMicrons”:10.0,“maxAreaMicrons”:150.0, “threshold”: 4.0, “watershedPostProcess”:true,“cellExpansionMicrons”:0.0, “includeNuclei”:true, “smoothBoundaries”: true, “makeMeasurements”: true}’);*saveDetectionMeasurements(‘/D:/………’) “*.

To determine the distribution of Iba1^+^ cells, Nearest Neighbor Distance (NND) analyses were performed using Fiji software’s NND plugin. Briefly, Iba1^+^ cells marked images were opened in Fiji and scaled accordingly. Firstly, images were converted to 8-bit, and a threshold was applied to isolate the white dots representing Iba1^+^ cells. The Analyse Particles function was run with default settings, followed by the execution of the NND plugin^74^.

### Immunoblotting analyses of blood plasma protein samples and protein samples isolated from brain FFPE tissue sections

Blood samples were collected and centrifuged at 15,000 g at 4 °C for 10 min to isolate plasma which was diluted at a 1:10 ratio with Laemmli buffer supplemented with 1mM PMSF. The samples were sonicated for 10 s (Sonoplus, Bandelin) and incubated at 95 °C for 5 min. Protein concentrations were determined using the Bio-Rad DC Protein Assay (Bio-Rad). A total of 20 µg of protein from each sample (except for CMH samples for TIMP-1 detection, where 40 µg was used) was mixed with 4× sample buffer at a 1:3 ratio and heated at 95 °C for 5 min before separation by SDS-PAGE and transfer onto PVDF membranes. Membranes were stained with Ponceau S solution (0.1% w/v Ponceau S dye in 5% v/v glacial acetic acid and dH_2_O) for 15 min on a rocker, and excess stain was removed by rinsing with distilled water before capturing images. Membranes were washed with 0.2% Tween-20 in PBS (3 × 10 min) and blocked with 5% milk in 0.1% Tween-20 PBS for 1 h at room temperature. Primary antibody incubation was performed overnight at 4 °C using fibrinogen (1:1000, Abcam, ab34269), MMP-9 (1:1000, Abcam, ab 38898) and TIMP-1 (1:1000, Proteintech, 16644-1-AP) antibodies. Following additional washes (3 × 15 min with 0.1% Tween-20 in PBS), membranes were incubated with appropriate HRP-conjugated secondary antibodies for 1 h at room temperature followed by washing. Chemiluminescent signals were developed using the Pierce™ ECL Western Blotting Substrate (Thermo Scientific, 32106) and Western Lightning^R^ Plus ECL (Revvity, NEL105001EA) and detected using X-ray film. To develop TIMP-1 signal, SuperSignal™ West Femto and SuperSignal™ West Pico PLUS substrates (Thermo Scientific) were mixed at a ratio of 1:15 (v/v) immediately before use. To analyze Western blot results, films were scanned and processed using Fiji software with the “Gel” analysis function. Images were first converted to 8-bit format and inverted, followed by background subtraction. A rectangle tool was used to define a single frame capturing the protein band of interest, ensuring the same frame dimensions were applied to all lanes. The first lane was labeled as “1” using the “Select First Lane” option, and subsequent lanes were labeled sequentially using the “Select Next Lane” option followed by Integrated Density was measured for each lane. For normalization, albumin density measurements, the most prominent band of the Ponceau stain, were taken. The corresponding treatment and control samples were always run in the same blot, and the relative quantification results of the treatment and control groups were only compared for individual blots, but not between blots.

To perform an automated capillary-based protein immunoassay, protein samples were isolated from the hippocampal region using FFPE tissue sections and the Qproteome FFPE Tissue Kit (Qiagen, 37623) following the manufacturer’s instructions. Fibrinogen protein level analysis was performed using the JESS capillary-based instrument (ProteinSimple, Bio-Techne) according to the manufacturer’s protocol. For normalization, a total protein assay (DM-TP01; ProteinSimple, Bio-Techne) was conducted as per the manufacturer’s instructions.

### Statistical analysis

Statistical analyses were performed using GraphPad Prism (v 8.3) software. Data sets were analyzed using an unpaired t-test, and summary data were presented as mean ± standard deviation. Statistical significance was indicated as * *p* < 0.05. Supplementary Table S3 summarizes sample sizes and p-values of the statistical tests for each analysis. All animals and samples were included in the analyses; no data or animals were excluded.

## Supplementary Information

Below is the link to the electronic supplementary material.

Supplementary Material 3 

Supplementary Material 1 

Supplementary Material 2

Supplementary Material 4

Supplementary Material 5

Supplementary Material 6

Supplementary Material 7

## Data Availability

Data is available as supplemental figures and tables. The microscopy imaging data presented in the study are openly available at https://doi.org/10.22029/jlupub-18791. Additional raw data supporting the conclusions of this article will be made available by the authors on request.
