# Supplementary material for: Hypoxia or tobacco-smoke exposure induce region-specific microvascular remodeling in the brain
Source: Sci Rep. 2026 Apr 17;16:12722. doi: 10.1038/s41598-026-45975-3 (PMC13090336; doi:10.1038/s41598-026-45975-3)

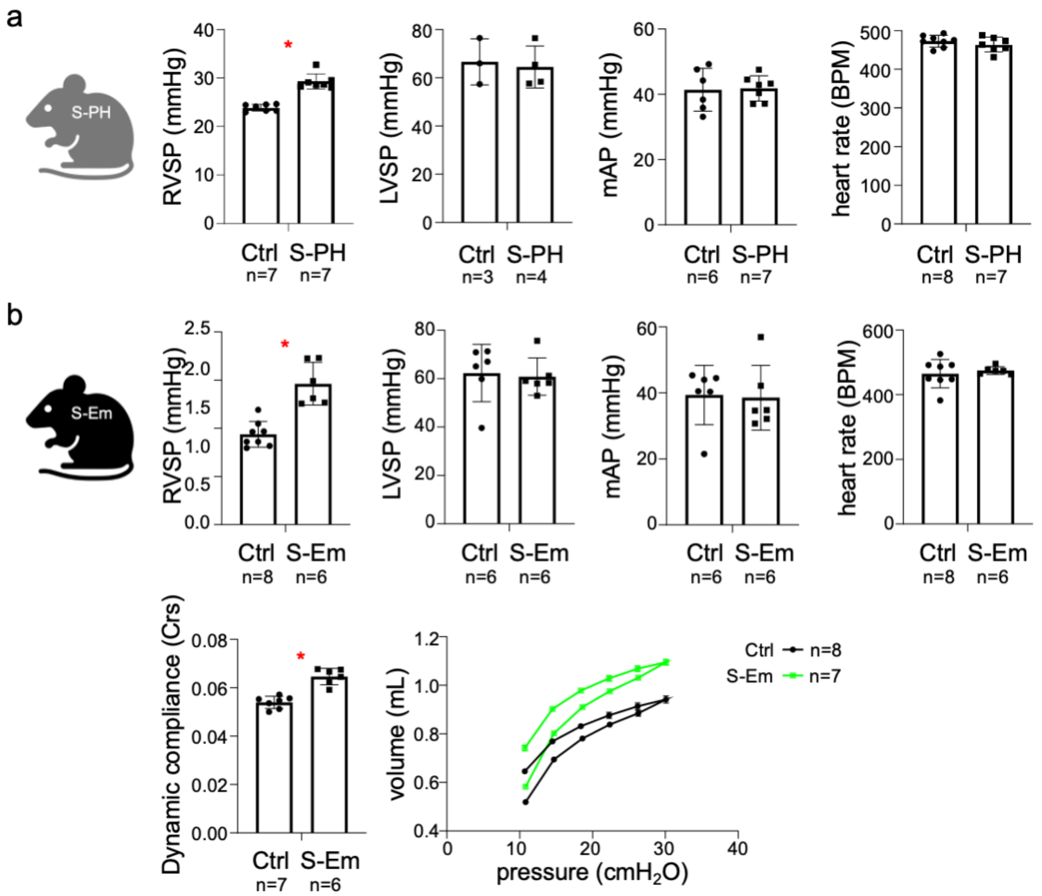

Figure S1. Respiratory and cardiovascular features of mouse models of chronic mild hypoxia and smoke exposure. Measurements (a) in the S-PH model (cigarette smoke exposure for 3 months) and (b) in the S-Em model (cigarette smoke exposure for 8 months) from the left: right ventricular systolic pressure (RVSP); left ventricular systolic pressure (LVSP); mean arterial pressure (mAP); heart rate of the animals during the *in vivo* hemodynamic measurements (beats per minute, BPM). Results of dynamic and static lung compliance measurements in S-Em mice indicating the development of emphysema are shown in the bottom panels. Unpaired t-tests $p<0.05$ are marked with a red asterisk.

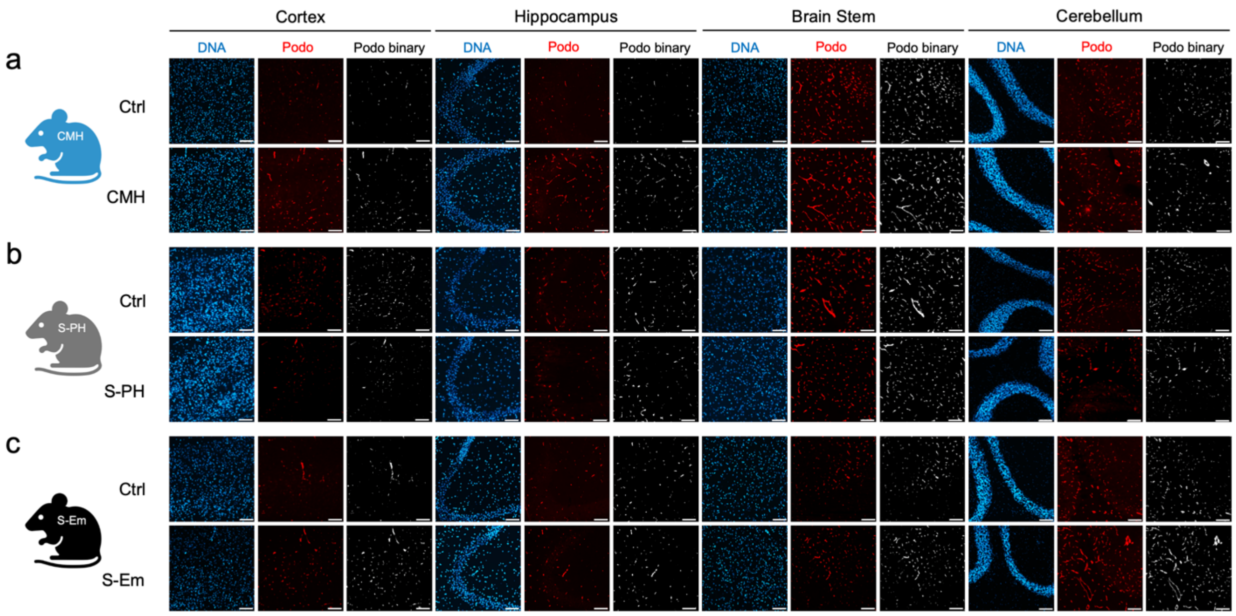

Figure S2. Endothelial marker immunofluorescence staining in mouse models of chronic mild hypoxia and smoke exposure. Examples of podocalyxin immunofluorescence staining of 5µm FFPE sections (**a**) in the CMH model (treated with 10% O_2 for 28 days), (**b**) in the S-PH model (treated with cigarette smoke for 3 months) and (**c**) in the S-Em model (treated with cigarette smoke for 8 months). Representative cortex, hippocampus, brain stem and cerebellum ROIs with DAPI (DNA, in blue) staining, podocalyxin staining (Podo, in red), and binary masks of podocalyxin staining (Podo binary, in white) are shown. Scale bars indicate 100 µm.

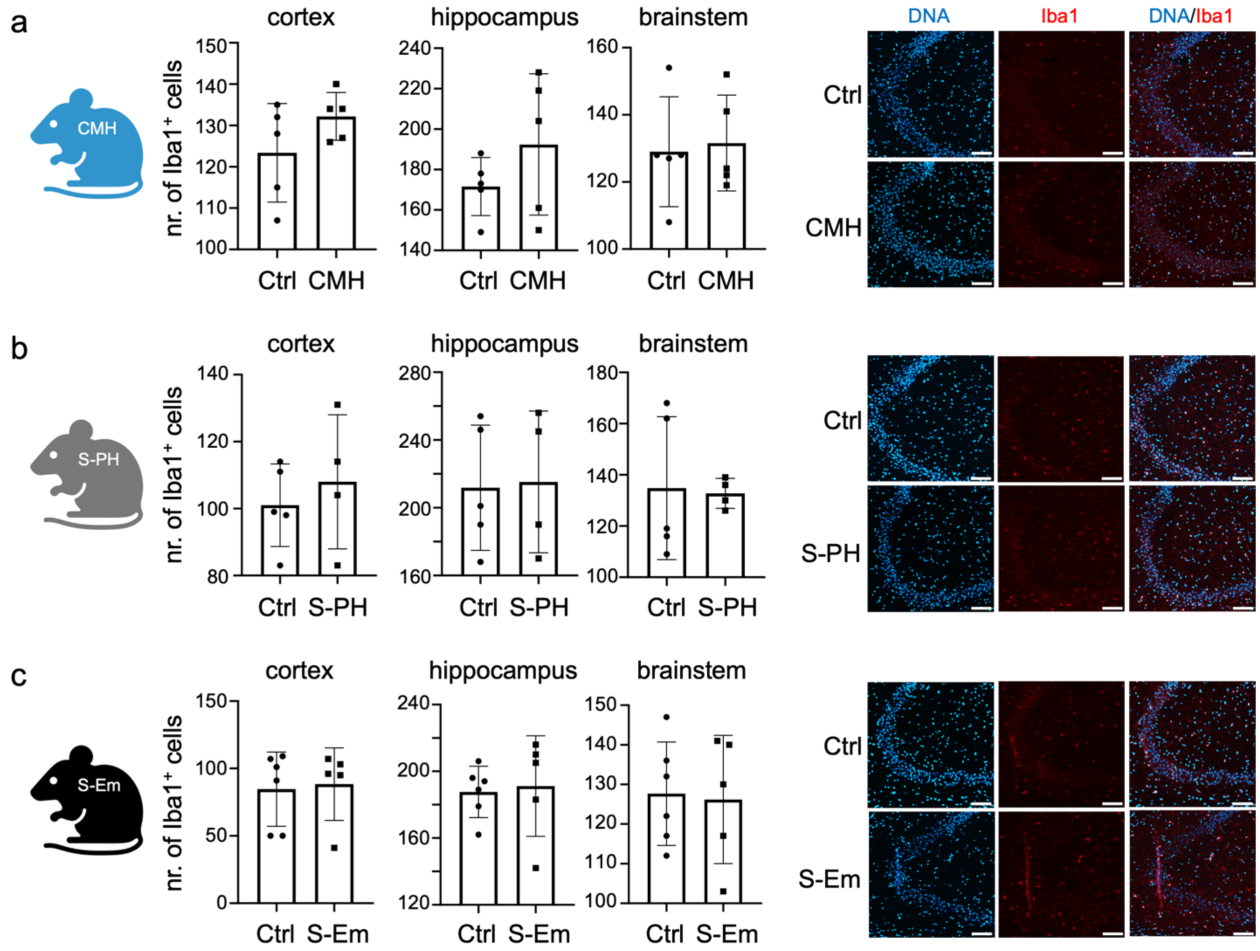

Figure S3. Microglia count in different brain regions in mouse models of chronic mild hypoxia and smoke exposure. Quantitative evaluation of microglia by counting Iba1$^+$ cells after immunofluorescence staining in equal-sized ROIs in control and treatment groups (**a**) in the CMH model (n=5 mice for both control and for the cohort treated with 10% O_2 for 28 days), (**b**) in the S-PH model (n=5 mice for the control and n=4 mice for the cohort treated with cigarette smoke for 3 months) and (**c**) in the S-Em model (n=6 mice for the control and n=5 mice for the cohort treated with cigarette smoke for 8 months). Representative hippocampus ROIs with DAPI (DNA, in blue) staining, Iba1 staining (in red) are shown. Scale bars indicate 100 µm.

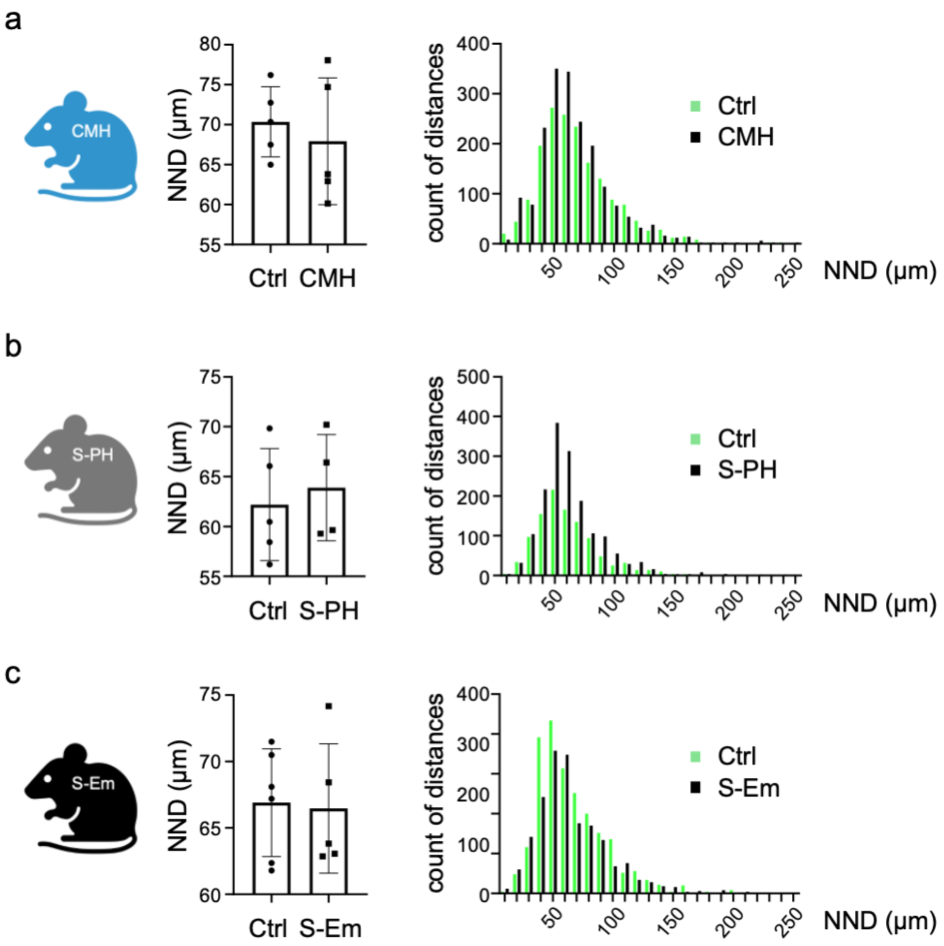

Figure S4. Microglia distribution in hippocampal brain regions in mouse models of chronic mild hypoxia and smoke exposure. Quantitative evaluation of microglia (Iba1$^+$ cells) distribution after immunofluorescence staining in equal-sized ROIs in control and treatment groups. Nearest neighbor distances (NND) were measured as described in the Materials and Methods. The data points on the left represent the average NND values for each animal. The bar graphs on the right visualize the same data set by showing the count of distances subdivided into 10 µm bins (**a**) in the CMH model (n=5 mice for both control and for the cohort treated with 10% O_2 for 28 days), (**b**) in the S-PH model (n=5 mice for the control and n=4 mice for the cohort treated with cigarette smoke for 3 months) and (**c**) in the S-Em model (n=6 mice for the control and n=5 mice for the cohort treated with cigarette smoke for 8 months). Unpaired t-test p values are shown.

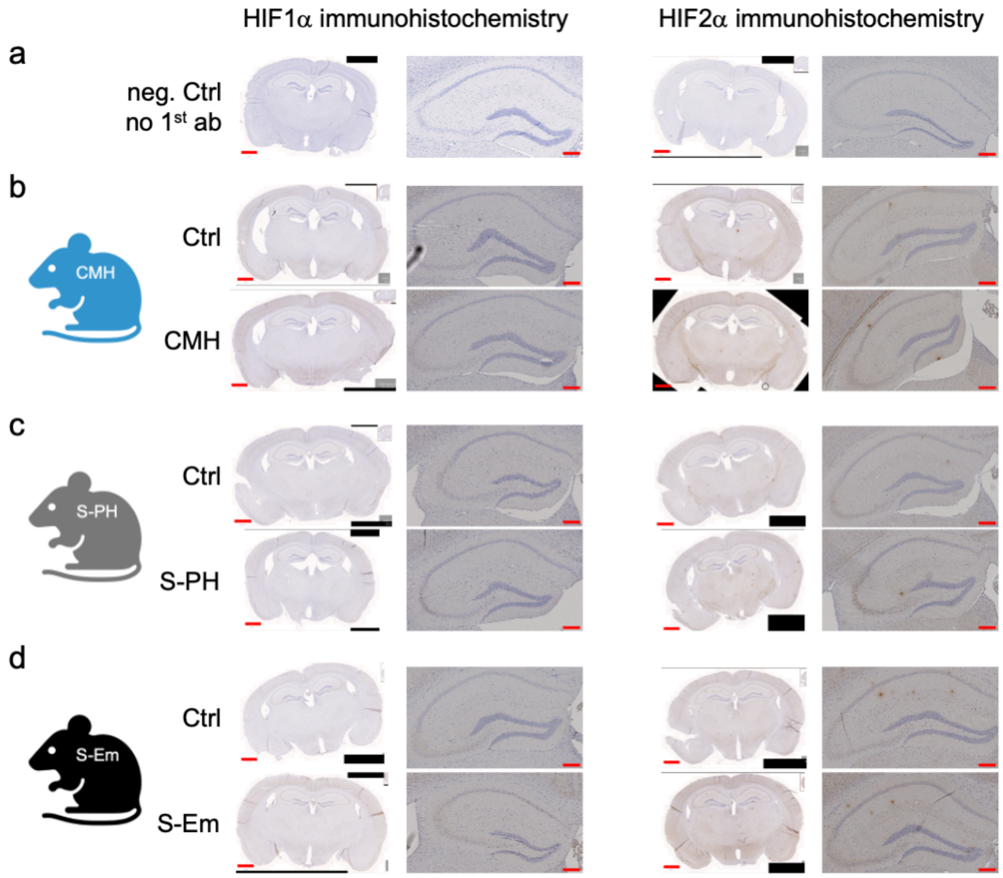

Figure S5. Immunohistochemistry staining of HIFs in mouse models of chronic mild hypoxia and smoke exposure. Representative examples of immunohistochemistry staining of HIF1a (left) and HIF2a (right) in 5 µm FFPE coronal mouse brain sections containing hippocampal regions. Zoom ins of the hippocampal regions are also shown. (**a**) Negative control staining without primary antibodies. HIF immunohistochemistry staining (**b**) in the CMH model (treated with 10% O_2 for 28 days), (**c**) in the S-PH model (treated with cigarette smoke for 3 months) and (**d**) in the S-Em model (treated with cigarette smoke for 8 months). Scale bars in red indicate 1 mm at the bottom left in the entire sections and 250 µm at the bottom right in the zoom ins.

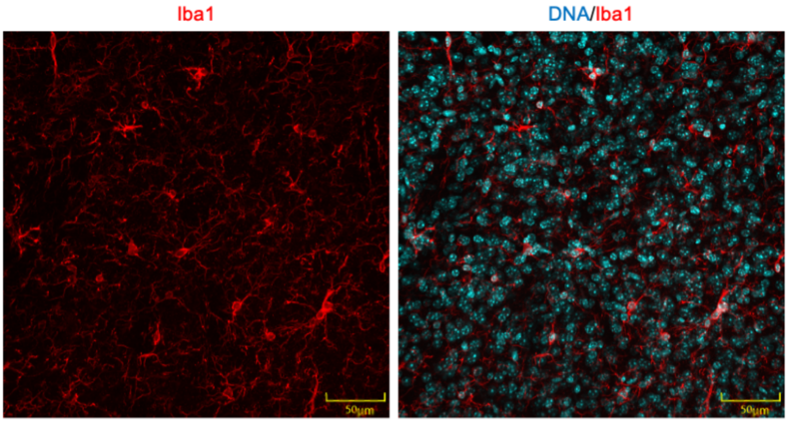

Figure S6. Microglia staining in 250 µm thick sections in the mouse models of smoke exposure. Exemplary Iba1 staining in 250 µm thick sections in the S-Em model. Z-projections of serial confocal images of DAPI (DNA) staining and microglia marker staining (Iba1) are shown. Scale bars indicate 50 µm.

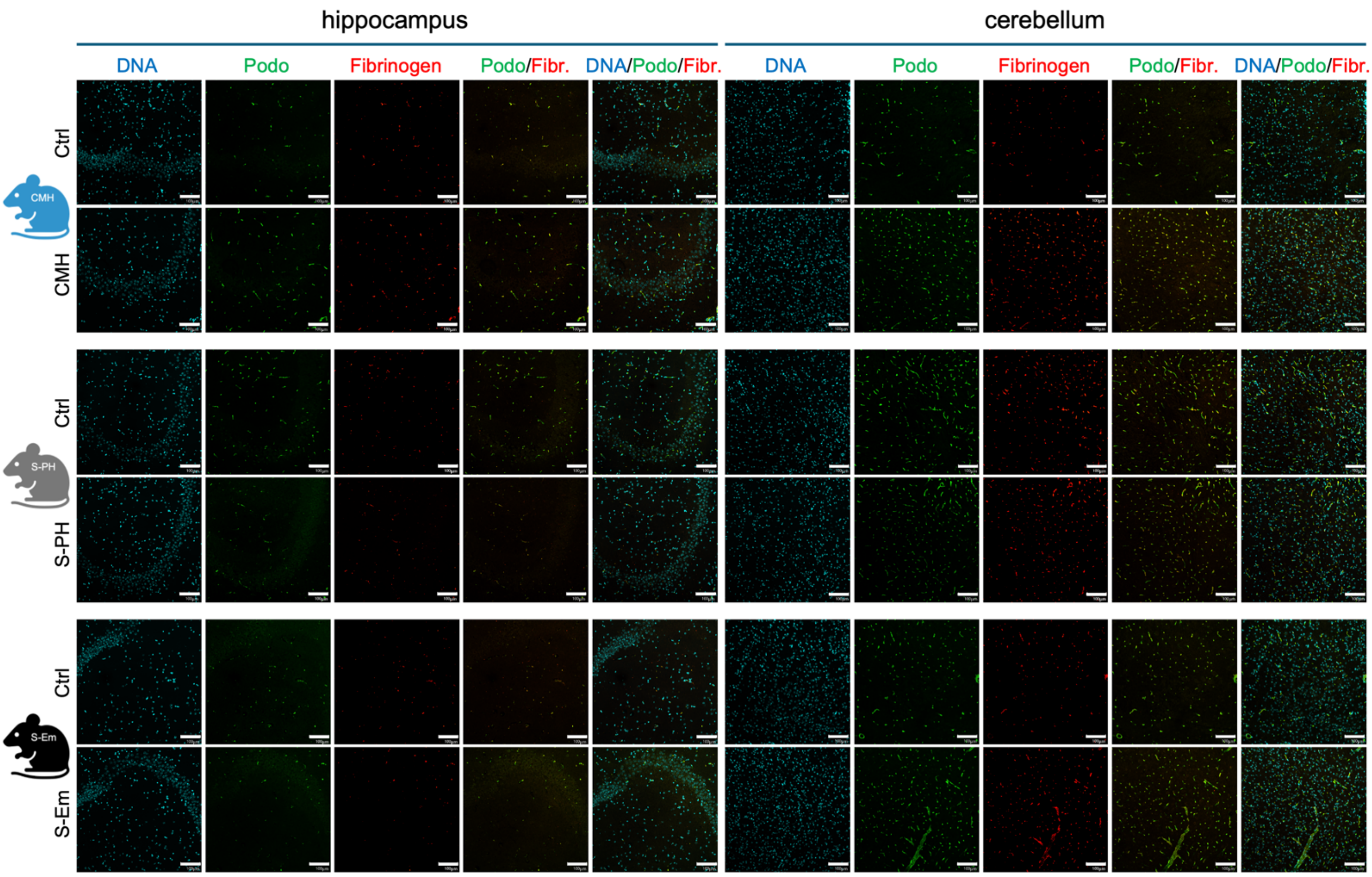

Figure S7. Vascular leakage marker fibrinogen and endothelial marker podocalyxin co-staining in 5 µm FFPE sections in mouse models of chronic mild hypoxia and smoke exposure. Examples of podocalyxin/fibrinogen immunofluorescence co-staining of 5 µm FFPE sections in the CMH, S-PH and S-Em models. Representative hippocampal and cerebellar ROIs with DAPI (DNA), podocalyxin (Podo) and fibrinogen (Fibr.) staining are shown.

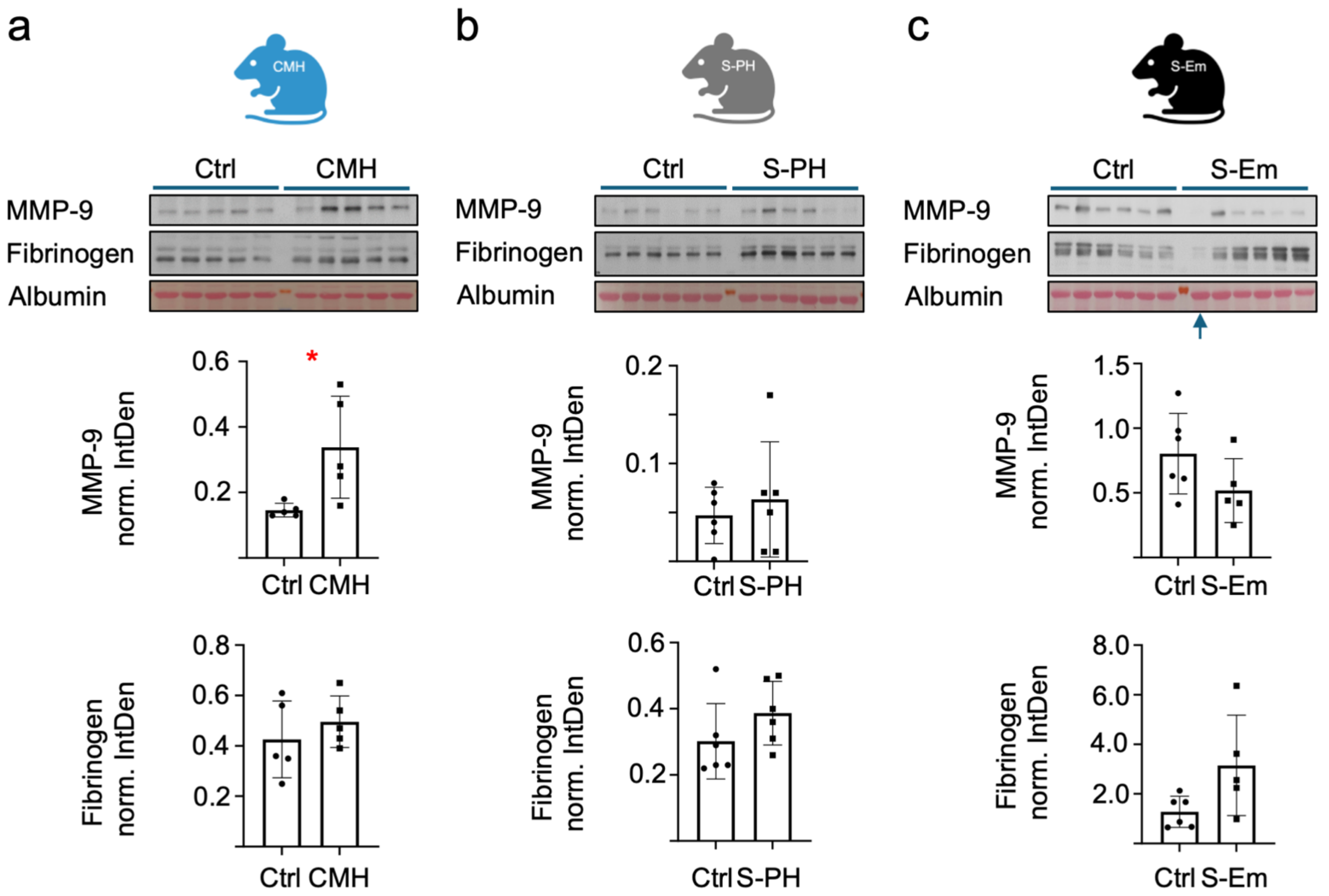

Figure S8. Western blot analyses of fibrinogen and MMP-9 in mouse models of chronic mild hypoxia and smoke exposure. Western blot and quantitative evaluation of fibrinogen and MMP-9 protein levels of blood plasma samples. The samples used in the immunoblots shown in Figure 6a-c were randomized and analyzed in a separate run. (**a**) CMH model (n=5 mice for both control and for the cohort treated with 10% O_2 for 28 days), (**b**) S-PH model (n=6 mice for both the control and for the cohort treated with cigarette smoke for 3 months) and (**c**) S-Em model (n=6 mice for the control and n=5 for the cohort treated with cigarette smoke for 8 months; the sample marked with the arrow was excluded from the analysis because the detection of fibrinogen and MMP9 was impaired). The integrated densities of the immunodetections of fibrinogen and MMP-9 were normalized to the albumin densities measured after Ponceau staining and are given as norm. IntDen. Unpaired t-tests $p<0.05$ is marked with a red asterisk.

Table S1. Vessel density data in mouse models of chronic mild hypoxia and smoke exposure. Vessel densities were measured in each ROI by quantitative immunofluorescence evaluation of the endothelial marker podocalyxin staining of 5µm FFPE sections as described in the Materials and Methods.

Table S2. Quantitative evaluation of immunohistochemistry staining of HIFs in mouse models of chronic mild hypoxia and smoke exposure. Single, double and triple positive cell count of immunohistochemistry staining of HIF1a and HIF2a in 5 µm FFPE mouse brain coronal sections containing hippocampal regions. Signal intensities were measured and categorized as described in the Materials and Methods.

Table S3. Sample sizes and p-values of statistical tests. Summary of statistical data for each variable measured, shown in the graphs in the Figures.

Video S1. Three-dimensional rotating view of microglia staining. Microglia structures illuminated by Iba1 immunostaining of a 250 µm brain tissue section of an S-Em mouse. The 3D rotating view corresponds to the Z-projection shown in Supplementary Figure S5.

Video S2. Three-dimensional integrated rotating view of endothelial marker podocalyxin and vascular leakage marker fibrinogen co-staining. Podocalyxin and fibrinogen combined immunofluorescence staining of a 250 µm brain tissue section with cerebellar region of an S-Em mouse.

Video S3. Z-stack image series of endothelial marker podocalyxin and vascular leakage marker fibrinogen co-staining. Podocalyxin and fibrinogen combined immunofluorescence staining of a 250 µm brain tissue section with cerebellar region of an S-Em mouse. The image series corresponds to the 3D rotating view shown in Supplementary Video S3.

Uncropped blot images 93

Salik et al Fig 6

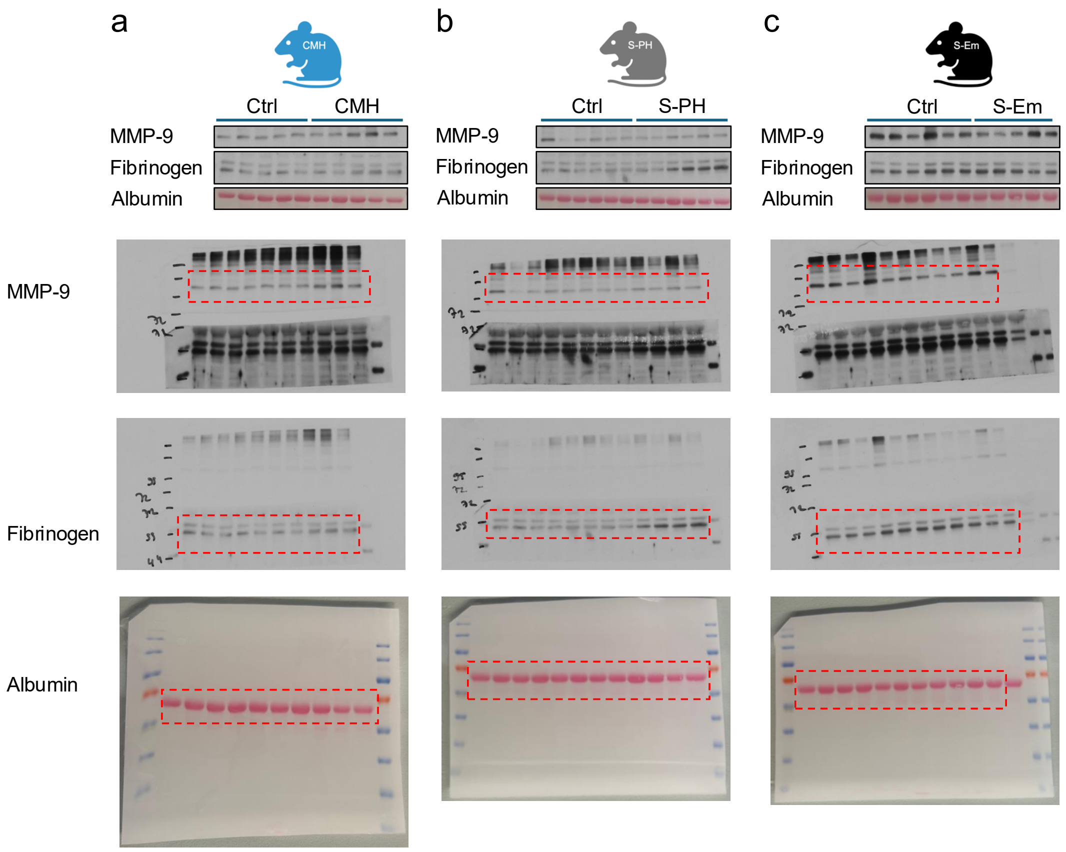

94

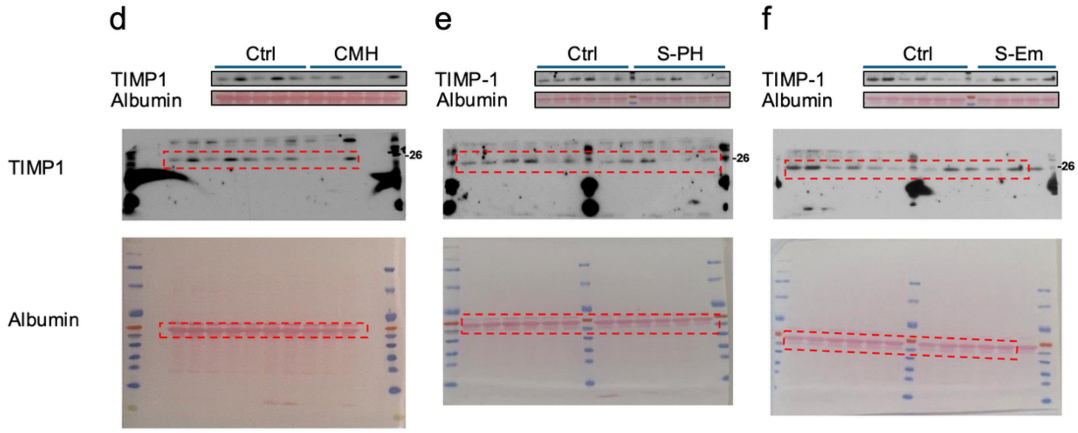

95

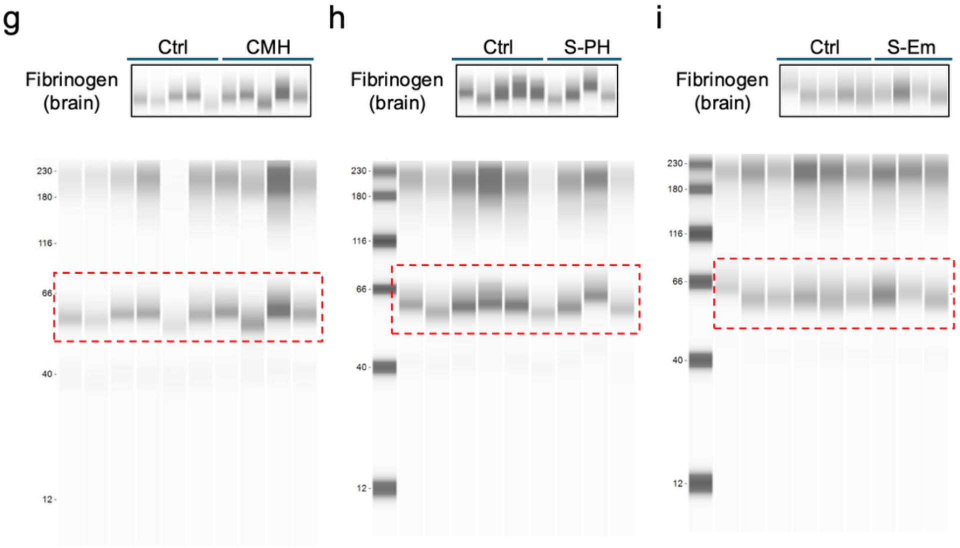

Salik et al Fig S8

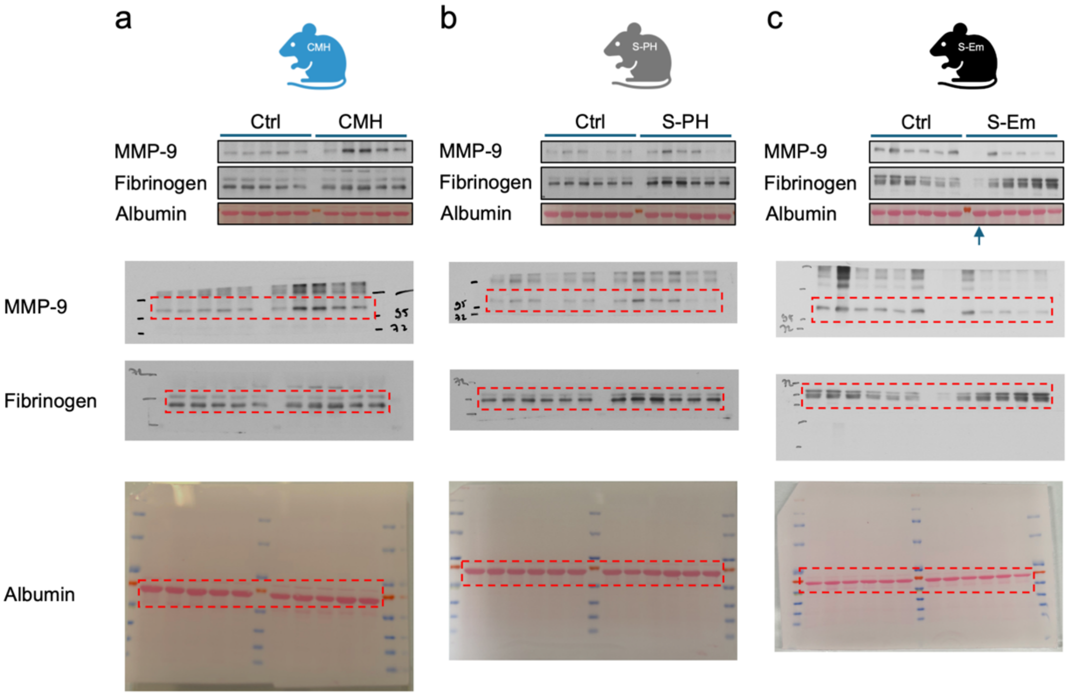

Supplement: Supplementary file 7 — Supplementary Material 7 [file 41598_2026_45975_MOESM7_ESM.pdf]
